# Facies and depositional environments of the Upper Muschelkalk (Schinznach Formation, Middle Triassic) in northern Switzerland

**DOI:** 10.1007/s00015-019-00340-7

**Published:** 2019-04-03

**Authors:** Arthur Adams, Larryn W. Diamond

**Affiliations:** Institute of Geological Sciences, University of Bern, Baltzerstrasse 1 + 3, 3012 Bern, Switzerland

**Keywords:** Upper Muschelkalk, Homoclinal ramp, Middle Triassic, Facies associations, Tempestites

## Abstract

Subsurface sedimentary strata in northern Switzerland, such as the Middle Triassic Upper Muschelkalk, are attracting interest as potential reservoirs for CO_2_ sequestration and for geothermal energy production. Characterizing facies in such strata aids prediction of reservoir properties in unexplored areas. Although well studied elsewhere, the Swiss Upper Muschelkalk has received little attention despite containing the southern-most deposits of the Central European Basin. The Upper Muschelkalk represents the deposits of a storm-dominated, homoclinal carbonate ramp, developed during a basin-wide 3rd-order transgressive–regressive cycle. Our facies analyses of nine boreholes across northern Switzerland reveal 12 lithofacies, eight lithofacies associations and four types of metre-scale 5th-order cycles corresponding to at least 23 short orbital eccentricity cycles. During the 3rd-order transgression, crinoidal bioherms developed across Switzerland followed by deep-ramp environments. Subsequently, tempestites were deposited up to and after the basin-wide maximum flooding surface. Lateral tempestite correlations indicate that Switzerland lay within an open-marine, mid-ramp environment during almost half of the depositional history. Mid-ramp deposits pass upwards to prograding shelly shoals, which sheltered a back-shoal lagoon containing patchy oolitic shoals. At the top of the Upper Muschelkalk, back-shoal sediments give way to coastal sabkha facies, which were overlain by oolitic shoals during a marine transgression. Shortly thereafter the top of the Upper Muschelkalk was dolomitized by brines from an overlying hypersaline environment that was later removed by a basin-wide erosive event. Overall, the paucity of porous shoal facies, unlike in southern Germany, has resulted in poor primary reservoir properties in the Upper Muschelkalk of Switzerland.

## Introduction

Carbonate sedimentary rocks commonly offer potential as aquifers for groundwater and as reservoirs for hydrocarbons, gas-storage and geothermal energy. The present-day rock-matrix properties of such reservoirs are largely determined by inheritance from the initial sedimentary and early diagenetic systems. Therefore, in addition to reconstructing later diagenetic and tectonic overprints, understanding and predicting reservoir properties requires understanding the carbonate depositional systems themselves, including characterising their vertical and lateral facies transitions, sedimentary stacking patterns and depositional morphologies (Read 1985; Burchette and Wright 1992; Ruf and Aigner 2004; Borkhataria et al. 2005; Palermo et al. 2010). All these features result from the interplay of factors influencing the early evolution of carbonate environments, including tectonic regime, climate, palaeobathymetry, biological assemblages, seawater chemistry and sediment production (Read 1985; Burchette and Wright 1992; Pomar 2001; Pomar and Hallock 2008).

When carbonate depositional systems develop during periods of uniform sedimentation in low tectonic-activity settings with gentle palaeoslopes, such as in intracratonic basins, they build homoclinal ramps (Read 1985; Burchette and Wright 1992). Such ramps are characterised by low slope gradients (< 1°), by an absence of a major slope break between the shoreline and basin, and by gradual facies transitions from sabkha deposits adjacent to lagoonal facies to shoals and mid- to distal-ramp facies below the storm wave-base (SWB) (Aigner 1985; Read 1985; Burchette and Wright 1992). These gradual facies transitions allow for the regional-scale predictability of reservoir properties (Borkhataria et al. 2005; Koehrer et al. 2010; Palermo et al. 2010). However, due to their low-angle morphologies, homoclinal ramp facies are particularly susceptible to early reservoir modification by multiple diagenetic environments (Read 1985; Burchette and Wright 1992, Pomar and Ward 1999; Adams and Diamond 2017). The resulting reservoir heterogeneities are therefore often directly related to the facies and depositional evolution of the carbonate ramp.

The facies of the Middle Triassic Upper Muschelkalk correspond well to a low-angle carbonate ramp (Aigner 1985). Studies in the Netherlands and south-western Germany demonstrate that the Upper Muschelkalk transitions from near-shore sabkha deposits to a low-energy lagoon, protected from open marine conditions by shelly/ooid barriers, to mid-ramp and distal-ramp storm-dominated sediments (Aigner 1985; Schauer and Aigner 1997; Borkhataria et al. 2005; Palermo et al. 2010). Although the reservoir properties of the Upper Muschelkalk of Germany are predictable and have been well studied (Braun 2003; Ruf and Aigner 2004; Koehrer et al. 2010; Palermo et al. 2010), the reservoir properties of the Upper Muschelkalk of Switzerland are spatially heterogeneous (Chevalier et al. 2010). This is in part due to its complex early diagenetic history (Adams and Diamond 2017), to the dolomitization of the Upper Muschelkalk (Adams et al. 2019) and to the burial history of the unit (Aschwanden et al. 2019). These studies have demonstrated that the bioclastic beds and calcitic mudstones of the Upper Muschelkalk generally have poor reservoir properties due to early cementation and compaction (Adams and Diamond 2017). However, the dolomitized mudstones of the Upper Muschelkalk can show good reservoir properties (Aschwanden et al. 2019), and some oolitic and shelly shoals also have porosities over a magnitude higher than mudstones (Adams and Diamond 2017), as is the case in the German Upper Muschelkalk (Braun 2003; Koehrer et al. 2010; Palermo et al. 2012). Despite the recognized connection between Upper Muschelkalk facies and reservoir properties, and in contrast to the many investigations of facies in the German Upper Muschelkalk, the Upper Muschelkalk of Switzerland has been the focus of only one descriptive facies study (Merki 1961). In light of the extensive deep-drilling campaigns by Nagra (National Cooperative for the Disposal of Radioactive Waste) and SEAG (Schweizerische Erdöl AG) since the publication of Merki (1961), the recent revelation of significant diagenetic differences between Switzerland and Germany (Adams and Diamond 2017; Adams et al. 2019), and the current interest in geo-energy applications of sedimentary reservoirs (Chevalier et al. 2010), a revaluation of the facies of the Swiss Upper Muschelkalk is warranted.

Accordingly, this study examines the facies and ramp evolution of the Middle Triassic Upper Muschelkalk of northern Switzerland, based on new investigations of Nagra and SEAG drill cores. The goals are to describe the lithofacies and lithofacies associations of the Swiss Upper Muschelkalk, to investigate its sequence stratigraphic framework, to reconstruct the ramp evolution and to compare the results with existing studies of the Upper Muschelkalk of southern Germany.

## Geology

### Palaeogeography

**Fig. 1:**
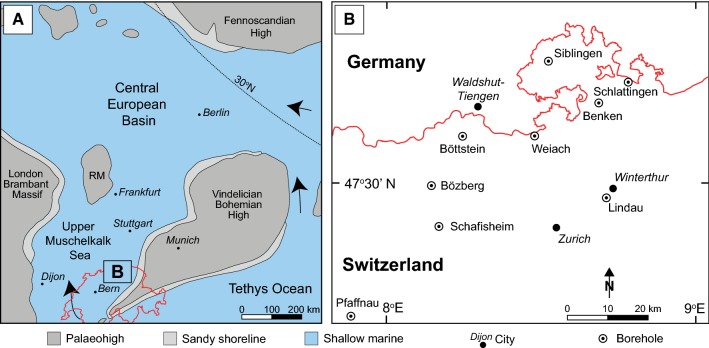
**a** Palaeogeography of the Central European Basin during the Middle Triassic, modified from Ziegler (1990). **b** Location of investigated boreholes in northern Switzerland

During the Anisian–Ladinian, the epicontinental Central European Basin (CEB) was a large, semi-enclosed, peripheral basin of the Tethys that extended from eastern France to eastern Poland and from Scandinavia to Switzerland (Ziegler 1990) (Fig. 1a, b). The CEB was enclosed by the Fennoscandian High in the north, the London Brabant Massif in the west and the Bohemian Massif/Vindelician High in the east. The basin was periodically connected to and restricted from the Tethys Ocean by the repeated opening and closing of three tectonically controlled gates (Szulc 2000). During periods of basin restriction, thick sequences of evaporites were produced (e.g., the Middle Muschelkalk and Keuper evaporites), while during periods of seawater connections, thick sequences of carbonates were produced e.g. the Lower Muschelkalk and Upper Muschelkalk (Aigner and Bachmann 1992).

The Upper Muschelkalk represents the deposits of a homoclinal carbonate ramp that formed during a Middle Triassic 3rd-order transgressive–regressive sequence (Aigner and Bachmann 1992). The ramp originated from the shorelines of the Vindelician High in eastern Switzerland and south-western Germany, and dipped north-westwards into the CEB (Aigner 1985). During the transgressive hemicycle in the southern CEB, crinoids immigrated into the Upper Muschelkalk Sea and formed metre-scale shoals and bioherms that rimmed the Vindelician High (Aigner 1985). When the Upper Muschelkalk Sea was at its maximum extension, the crinoidal shoals were drowned and overlain by thick nodular limestones (Aigner 1985; Franz et al. 2015). During the subsequent marine regression, the nodular limestones were overlain by repeated shallowing-upwards tempestite sequences that formed on the mid-ramp during monsoonal winter storms in an otherwise semi-arid basin (Parrish 1993). The mid-ramp was overlain by packstone–grainstone shoals that rimmed the Vindelician High (Aigner 1985). These shoals separated the tempestite-rich mid-ramp from a sheltered backshoal lagoon (Alesi 1984). Following the deposition of low-energy backshoal sediments, a basin-wide sea-level fall formed lenticular, nodular and chicken-wire sulphates, and desiccation cracks at the top of the Upper Muschelkalk (Schauer and Aigner 1997; Adams et al. 2019). In many regions in the south-eastern CEB, the final sulphate-rich facies of the Upper Muschelkalk have been eroded away during the deposition of the overlying brackish/terrestrial Lettenkohle unit (Franz et al. 2015). The nature of the last beds deposited prior to the Lettenkohle is therefore unknown.

### Lithostratigraphy

**Fig. 2:**
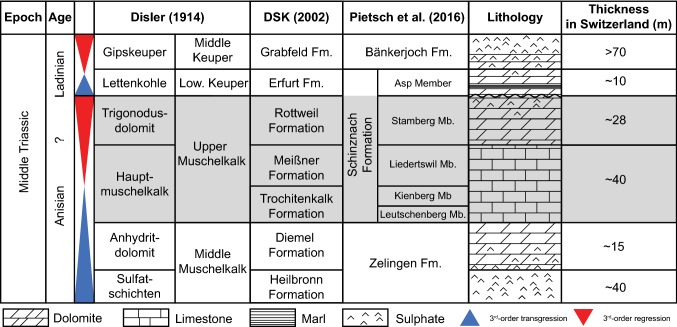
Lithostratigraphic context of the Upper Muschelkalk according to various authors. Shaded area: units examined in this study. Transgressive and regressive 3rd-order sequences are from Franz et al. (2015). The classifications of Disler (1914) and Pietsch et al. (2016) are used throughout this study. *DSK*: Deutsche Stratigraphische Kommission (2002)

Since the lithostratigraphic classification of Disler (1914), the Upper Muschelkalk has been divided into two subunits: the lower calcitic Hauptmuschelkalk and the overlying fully dolomitized Trigonodus Dolomit (Fig. 2). Following the HARMOS project (an effort to reclassify and unify Swiss lithostratigraphy; Stratsky et al. 2016), the Upper Muschelkalk was renamed the Schinznach Formation and subdivided into five members; the Leutschenberg, Kienberg, and Liedertswil Members (formerly Hauptmuschelkalk), the Stamberg Member (formerly Trigonodus Dolomit) and the Asp Member (formerly Lettenkohle) (Pietsch et al. 2016). Given our stated motivation to assess the carbonate ramp facies, the present study focuses on the Trigonodus Dolomit and Hauptmuschelkalk without detailed attention to the Lettenkohle facies, and therefore the term Schinznach Formation is not used. Instead, the nomenclature of Disler (1914) is applied (i.e. Upper Muschelkalk) along with the new member divisions for the Hauptmuschelkalk (Pietsch et al. 2016).

## Well core and methods

Approximately 466 m of drill core was examined from nine wells drilled in northern Switzerland by Nagra (National Cooperative for the Disposal of Radioactive Waste), SEAG (Schweizerische Erdöl AG) and the SBB (Swiss Federal Railways) (Fig. 1b). Following sample selection, each drill core, with the exception of Benken, was scanned and photographed at the University of Bern with a GEOTEK© Multi Sensor Core Logger (MSCL). Facies analysis was based on bed-by-bed logging of each drill core complemented by MSCL drill core images. The Benken drill core was logged based on a report by NAGRA (2001) and on thin sections and samples stored at the University of Bern.

A total of 275 thin sections from all core samples were examined at the University of Bern by conventional, plane-polarized transmitted light microscopy, UV-epifluorescence microscopy (UV-F) and hot-cathodoluminescence microscopy (CL) using a 20 kV beam as described by Ramseyer et al. (1989). Some thin sections were stained with a mixture of Alizarin Red S and potassium ferricyanide based on the method of Dickson (1966) to differentiate calcite from dolomite. The classification of carbonate textures follows the nomenclature of Dunham (1962).

## Facies analysis

**Fig. 3:**
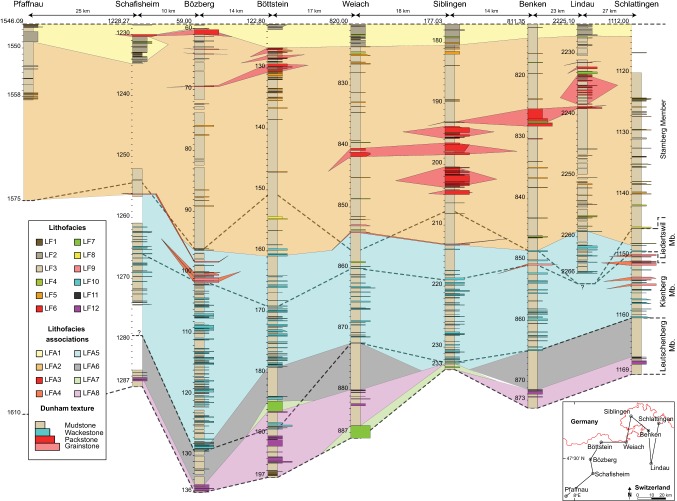
Regional cross-section composed of facies logs of boreholes from northern Switzerland. Lithofacies association correlation is based on cyclostratigraphic correlations. Member divisions of the Upper Muschelkalk after Pietsch et al. (2016) are marked with dashed lines. The Stamberg Member is equivalent to the dolomitized sediments of the former Trigonodus Dolomit, while the combined 3 other members are equivalent to the former Hauptmuschelkalk. Note that the Bözberg borehole was drilled at a 45° angle relative to the Upper Muschelkalk bedding and thus appears longer than its true vertical thickness

**Fig. 4:**
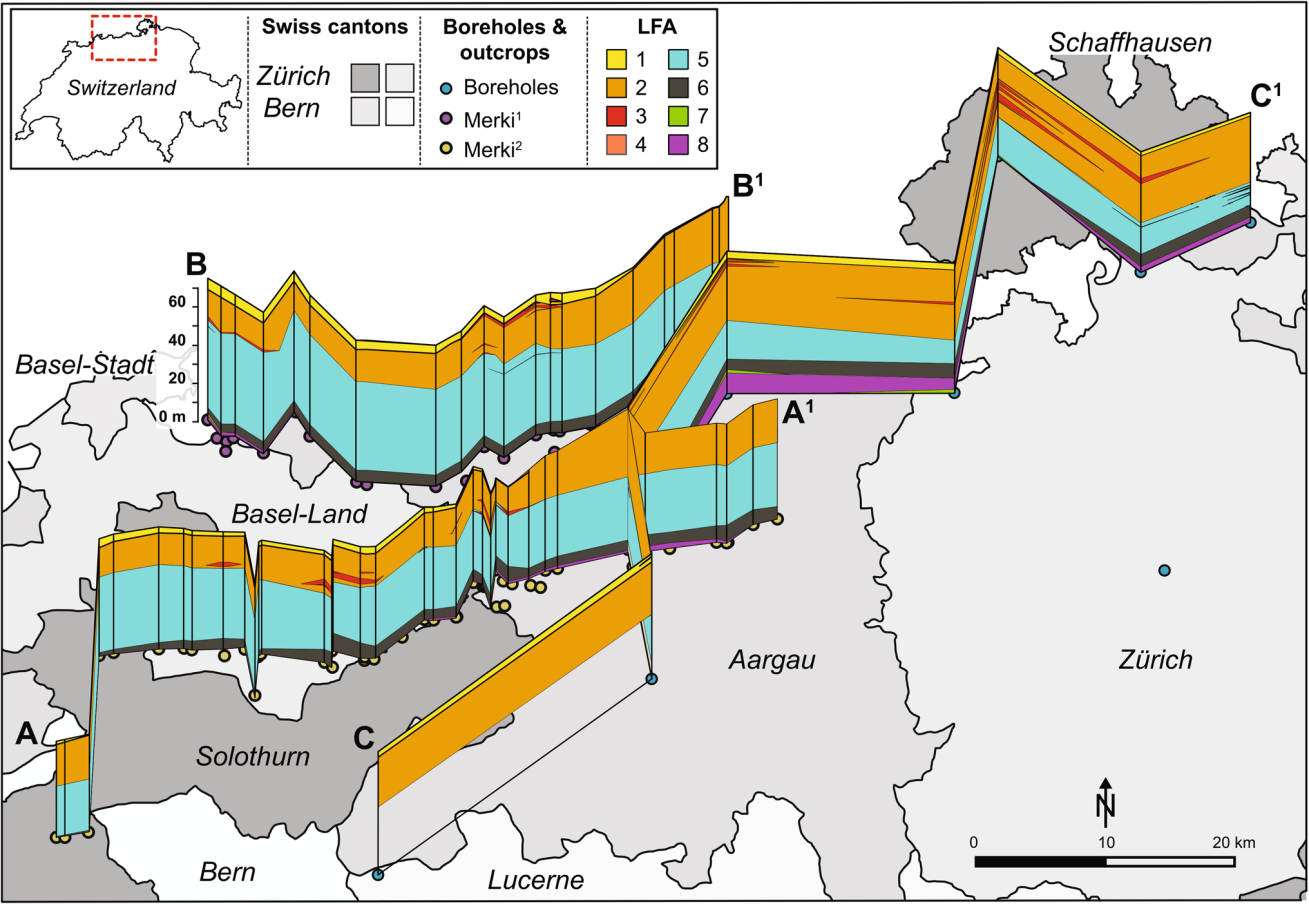
Fence diagrams of interpreted lithofacies associations (LFA) in northern Switzerland, created from borehole data from this study and outcrop investigations by Merki (1961). Cross-section A–A^1^ from Merki (1961; Tafel IV); cross-section B–B^1^ from Merki (1961; Tafel V); cross-section C–C^1^ from boreholes in this study (Fig. 3). Note that the Lindau borehole is not connected with the C–C^1^ fence due to practical constraints

The Upper Muschelkalk of northern Switzerland consists of 12 lithofacies (Table 1), which are grouped into eight genetically linked lithofacies associations (LFA) based on common Dunham (1962) textures, bioturbation, grain sizes, components and sedimentary structures (Figs. 3, 4). Facies and LFA described below are similar to the nearshore, backshoal, shoal, foreshoal and bioclastic tempestite facies and LFA identified in previous studies of the Upper Muschelkalk of south-western Germany (Alesi 1984; Aigner 1985; Braun 2003; Koehrer et al. 2010; Palermo et al. 2010; Warnecke and Aigner 2019b). The principal skeletal components of Upper Muschelkalk bioclastic facies are crinoid ossicles, bivalves, brachiopods, gastropods and bones. Non-skeletal components consist of oncoids, peloids, ooids, intraclasts, black pebbles and microsparitic matrix. Mudstones are the dominant facies of the Swiss Upper Muschelkalk. Each LFA is described and interpreted below in detail.

**Table 1:** Summary of defining characteristics of the 12 lithofacies distinguished in this study

Facies no.	Name	Sedimentary structures	Components^a^	Bioturbation	Bed thickness and sorting	Interpretation
1	Laminated dolomudstone	Parallel–wavy mm-scale laminations, wave-ripple laminations, chert nodules, sulphate nodules, flat-pebble conglomerates	Peloids (c), lithoclasts (u)	None	Centimetre thick beds; good sorting	Microbial laminates in an intertidal environment
2	Pelitic dolowackestone–packstone	Wavy laminations, sulphate nodules, massive bedding	Peloids (a), molluscs (c), lithoclasts (c)	None–moderate	Decimetre–several meter thick beds; poor sorting	Sheltered peritidal deposits
3	Massive–nodular (dolo)mudstone	Massive and nodular bedding, chicken-wire textures, evaporite nodules, breccias, faint laminations, marly sheets	Lithoclasts (c), skeletal debris (r), peloids (r), crinoids (r)	None–intense	Decimetre–several meter thick beds; good sorting	Low-energy subtidal lagoonal deposits
4	Lithoclastic dolowackestone–packstone	Normal grading, erosive sole	Black pebbles (c), lithoclasts (c), molluscs (c), peloids (c), bones (r)	Light	Centimetre–decimetre thick beds; poor sorting	Channel or event deposits in the subtidal lagoon environment
5	Bioclastic (dolo)wackestone–packstone	Poorly defined beds, mm–cm erosive scours	Bivalves (a), peloids (a), brachiopods (c), lithoclasts (c), bones (r)	Light, bored lithoclasts	Centimetre–decimetre thick beds, poor–moderate sorting	Shoal spillover lobes into the backshoal lagoon
6	Oolitic (dolo)wackestone–grainstone	Poorly defined beds, cross-bedding, horizontal laminations	Ooids (a), peloids (c), molluscs (c), lithoclasts (u)	None–light	Centimetre–decimetre thick beds, poor–moderate sorting	Ooid shoal complexes
7	Oncolitic (dolo)wackestone–packstone	Laminations created by horizontally aligned oncoids, erosive sole	Oncoids (a), ooids (a), molluscs (c), lithoclasts (c)	None	Centimetre–decimetre thick beds, well sorted	Oncolitic channel-fills
8	Bioclastic (dolo)packstone–grainstone	Horizontal laminations, normal grading, erosive sole, mouldic porosity	Shell debris (a), lithoclasts (c)	None	Centimetre–decimetre thick beds; moderate–good sorting	Proximal shoal spillover lobes
9	Shelly packstone–grainstone	Low-angle laminations, erosive scours, amalgamated bedding	Micritized shell debris (a), brachiopods (c), glauconite (c), crinoids (u), ooids (u), forams (r), crinoids (r), gastropods (r)	None	Decimetre-thick beds, sometimes amalgamated; good sorting	Shell dominated shoal bodies
10	Scoured skeletal wackestone–packstone	Planar/low-angle laminations, normal grading, hummocky-cross stratification, mm-cm erosive scours	Shell debris (a), crinoids (a), gastropods (c), intraclasts (c), forams (u), peloids (u)	None–light	Centimetre–decimetre thick beds; poor–moderate sorting	Tempestites
11	Laminated mudstone–wackestone	Planar laminations, mm-thick lag deposits, marly laminations	Crinoids (u)	None	Centimetre thick beds; good sorting	Distal tempestites
12	Crinoid dominated wackestone–packstone	Massive and nodular bedding	Crinoids (a), gastropods (a), peloids (c), intraclasts (u)	Moderate–intense	Centimetre–decimetre thick beds; poorly sorted	Crinoidal bioherm

^a^Component frequency: (a) abundant, (c) common, (u) uncommon, (r) rare

### LFA 1 peritidal carbonates

**Fig. 5:**
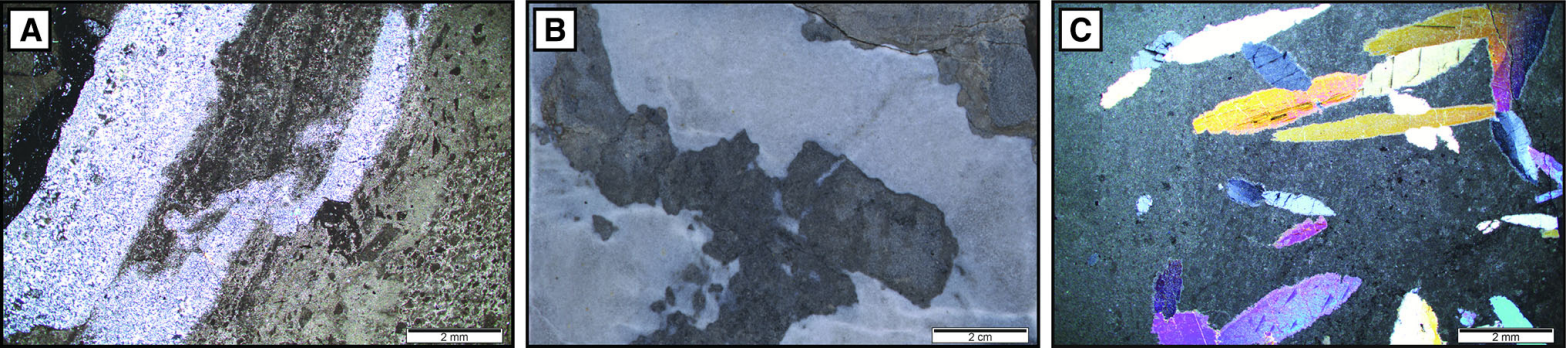
Drill core images and photomicrographs of thin sections under plane- and cross-polarized light. **a** Cross-polarized image of peloid-rich microbial laminates. The matrix is fully dolomitized and contains dark grey intraclasts, light grey peloids, silicified laminates in white and a stylolite bordering the silicified laminates in black. Pfaffnau 1546.12 m. **b** Drill core image of white chicken-wire anhydrite in a grey dolomitized matrix. Schafisheim, 1234.69 m. **c** Cross-polarized image of anhydrite laths. Pfaffnau, 1546.46 m

**Fig. 6:**
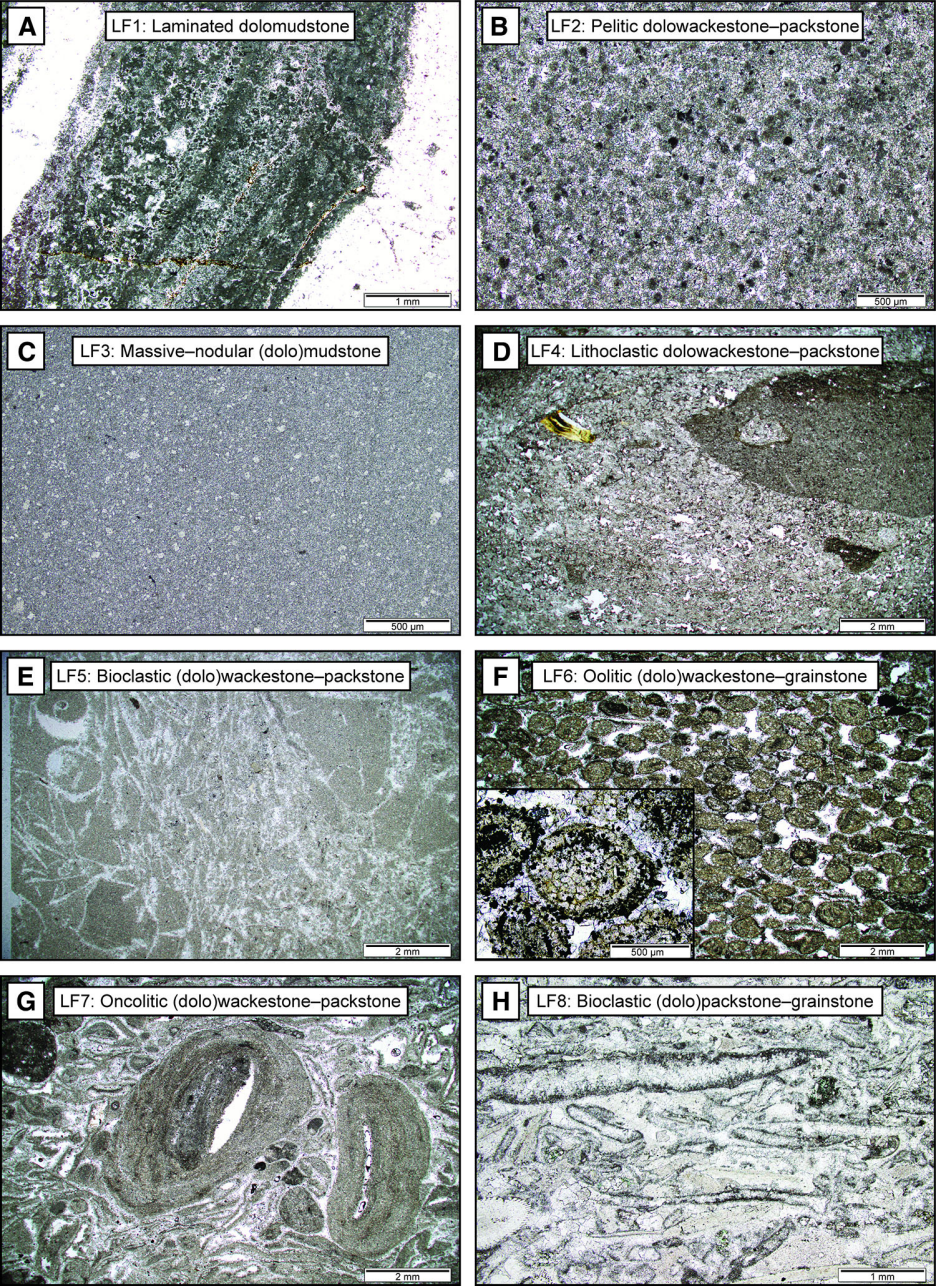
Photomicrographs of thin sections from lithofacies 1–8 under plane-polarized light. **a** Laminated dolomudstone (LF1) from Pfaffnau, 1546.12 m. White areas on left and right are chertified sediments. **b** Pelitic dolowackestone–packstone (LF2) from Schafisheim, 1231.47 m. **c** Massive–nodular (dolo)mudstone (LF3) from Schafisheim, 1285.34 m. White patches are Hauptmuschelkalk dolomites. **d** Lithoclastic dolowackestone–packstone (LF4) from Weiach, 853.73 m. Image contains a large (> 1 cm) lithoclast on the right and a yellow bone fragment in in left. **e** Bioclastic (dolo)wackestone–packstone (LF5). All bioclasts have been leached and only mould exist in the dolomitized sediments. **f** Oolitic (dolo)wackestone–grainstone (LF6) from Siblingen, 204.77 m. Inset of dolomitized ooid demonstrates that details of ooids are unrecognizable due to matrix dolomitization. **g** Oncolitic (dolo)wackestone–packstone from Benken, 827.55 m. Oncolites can reach multiple cm in length in LFA 7 at the base of the Upper Muschelkalk. **h** Bioclastic (dolo)packstone–grainstone from Schlattingen, 1147.95 m. All molluscs have been micritized, leached, cemented and fragmented, while crinoids (bottom left) have generally resisted these processes

*Description*: Lithofacies association 1 consists of the dolomitized uppermost 3–6 m of the Upper Muschelkalk and Stamberg Member. These dolomitized sediments contain primary and diagenetic evaporite textures such as lenticular anhydrite laths, chicken-wire anhydrite, anhydrite nodules and their dissolution vugs, and chert nodules and beds (Fig. 5). Dolomitization was fabric destructive; however, UV-fluorescence reveals that facies consist of microbial-laminated dolomudstone (*LF1*), pelitic dolowackestones to dolopackstones (*LF2*) and dolomudstone (*LF3*) (Fig. 6a, b, c). Microbial-laminated and pelitic sediments alternate frequently at the top of some boreholes (*Schafisheim* in Fig. 3). Anhydrite, microbial laminates, breccias and desiccation textures increase in frequency upwards towards the contact with the overlying Lettenkohle.

*Interpretation* Desiccation cracks, anhydrite laths and chicken-wire anhydrite indicate arid supratidal conditions at the end of the deposition of the Upper Muschelkalk. Alternations between microbial-laminated and pelitic sediments commonly occur in intertidal and supratidal environments along modern shallow and epeiric seas (Davies 1970; Shinn 1983). Chertified microbial-laminates are uncommon in modern supratidal settings; however, they are common features of ancient tidal flats (Shinn 1983). The upwards increase in evaporite textures and microbial laminated sediments results from increasing evaporite cementation during dolomitization (Adams and Diamond 2017; Adams et al. 2019) and an upward transition from peritidal to supratidal environments. Based on the sedimentary evidence, LFA 1 is interpreted to represent a regressive hypersaline peritidal–supratidal environment. Similar sediments of the German Upper Muschelkalk are also interpreted as peritidal–supratidal facies (Alesi 1984; Schauer and Aigner 1997; Braun 2003; Koehrer et al. 2010).

### LFA 2 sheltered backshoal deposits

*Description* This lithofacies association varies in thickness between 20 and 37 m, which makes it the thickest LFA of the Upper Muschelkalk. It is composed of massive–bioturbated dolomudstones (*LF3*), sparse cm–dm thick pelitic dolo-wackestones (*LF2*), and black pebble (*LF4*) (Fig. 6d) and bioclastic (*LF5*, *LF8)* (Fig. 6e) dolo-wackestones to dolo-grainstones. Mudstones are the dominant constituents. Bioclastic beds are normally graded, poorly sorted, show rare scoured soles and pass gradually into the overlying mudstones. Bioclasts are not always confined to bioclastic beds and are often found in the mudstones below and above bioclastic facies. Ooids are found in some bioclastic facies but mainly occur in metre-scale LFA 3 bodies within LFA 2. The base of LFA 2 is marked by the first regular appearances of shelly packstones–grainstones (*LF9*) and scoured skeletal sheets (*LF10*).

*Interpretation* The massive textures of *LF3* could be attributed to sediment homogenization by bioturbation, sedimentation below the fair-weather wave base (FWWB) or sedimentation in a sheltered environment. Although the heavy bioturbation of *LF3* could mask any evidence of wave activity, evidence of wave agitation is found neither in the non-bioturbated mudstones nor within bioclastic beds. Bioclastic facies in LFA 2 show none of the storm-associated structures of the Upper Muschelkalk, i.e. regular scoured soles, planar laminations, hummocky cross-stratification (HCS), or marl drapes (Aigner 1985), and these bioclastic facies also lack the characteristic crinoid-dominated bioclastic assemblages of mid-ramp Upper Muschelkalk tempestites (Aigner 1985; Koehrer et al. 2010; Palermo et al. 2010). Additionally, the presence of black pebbles suggests sedimentation in a near-shore environment (Strasser 1984). Since lithofacies association 2 was deposited between the peritidal carbonates of LFA 1 and the shoal facies of LFA 4, the mudstone-dominated sediments indicate that LFA 2 was deposited in a low-energy lagoon, sheltered from most large storms by ooid and shelly shoals (LFA 3 and 4). Moderate to strong bioturbation indicates that the sediments were well oxygenated and that waters were not hypersaline during most of their depositional history. This LFA is recognized in the southern German Upper Muschelkalk (Koehrer et al. 2010).

### LFA 3 ooid shoals/bars

*Description* Oolites (*LF6*) consist of metre-scale cross-bedded and massive dolo-wackestone to dolo-grainstones that occur at the top and middle of LFA 2 (Fig. 6f). Oolites form lenticular shaped bodies that are continuous over kilometres in east–west and north–south directions (Merki 1961). Individual oolitic grainstones are well sorted and form 20 to 100 cm thick beds, which are separated from one another by cm–dm thick ooid-rich mudstones-packstones. The oolites are composed of a mixture of micritized shelly-hash and well rounded, 1 mm diameter ooids (Fig. 6f). Based on the concentric arrangement of organic material (Fig. 6f inset) and rounded shape, these ooids may correspond to type-1 ooids after the classification of Strasser (1986). Within some oolites, thin beds of ooid-rich oncolitic dolo-wackestones to -packstones (*LF7*) occur (Fig. 6g). Ooids can also be found in bioclastic (dolo)packstones–grainstones (*LF8*) (Fig. 6h) but never in the same amount as in *LF6*. The bases of oolite beds are not scoured and Dunham textures gradually pass upwards from mud- to grain- to mud-supported fabrics.

*Interpretation* Dolo-packstone to -grainstone beds, cross-bedding, and the presence of ooids and micritized shell-hash indicate high-energy conditions. Repeated upwards textural changes from mudstones to grainstones and back to mudstones suggests lateral migration of the oolite shoals. The spatial distribution of LFA 3 shows that high-energy oolitic shoals occurred as a rim of isolated patches offshore from the Vindelician High in Switzerland, as well as in southern Germany (Alesi 1984; Aigner 1985). Adjacent to ooid bodies, towards the palaeoshoreline, oncoidal facies indicate deposition in tidal channels in the backshoal environment (Aigner 1985). Based on its facies characteristics and spatial distribution, LFA 3 is interpreted to represent ooid shoals rimming the Vindelician High.

### LFA 4 shoal/proximal ramp deposits

**Fig. 7:**
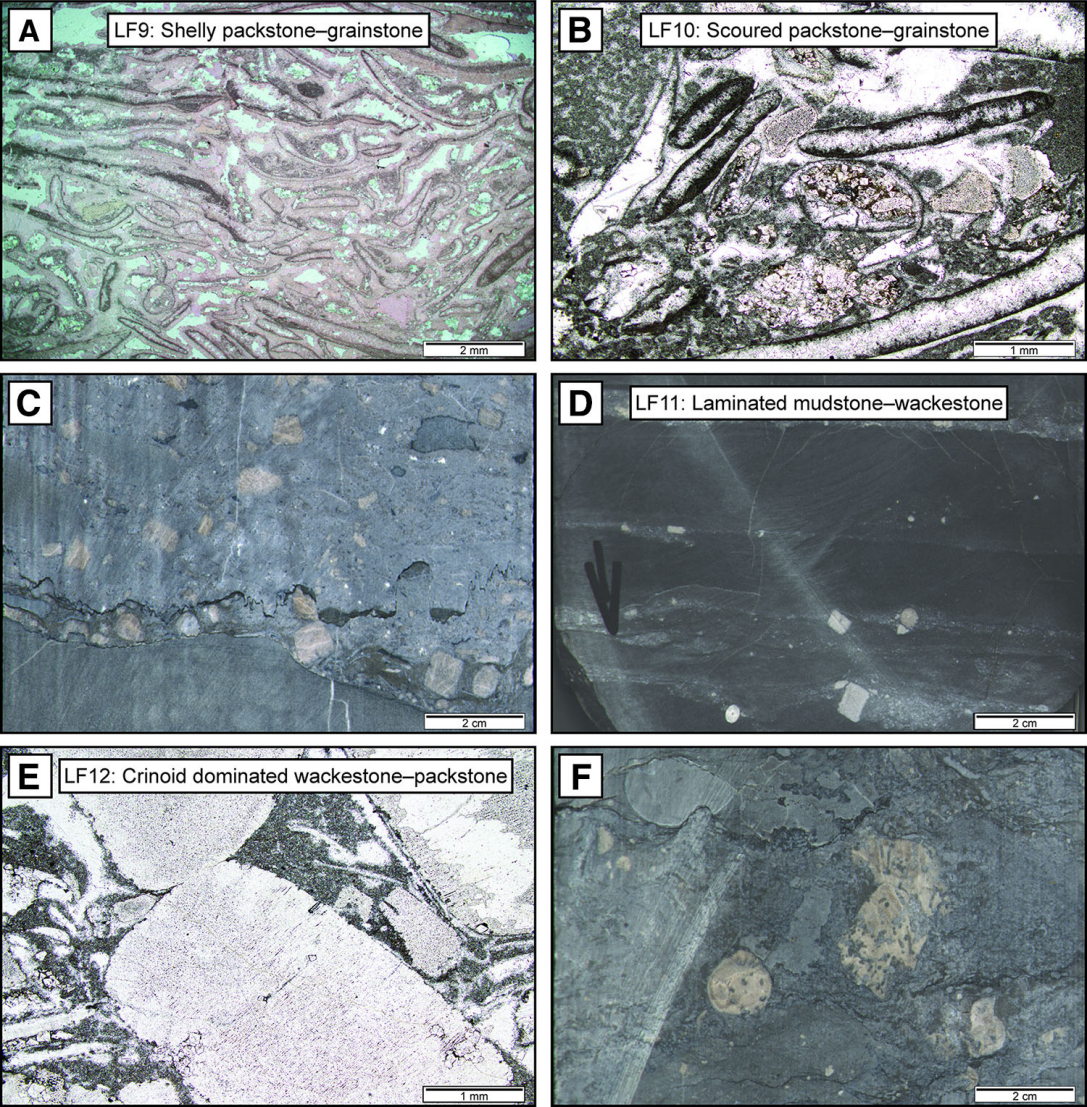
Photomicrographs of thin sections and drill core images from lithofacies 9–12 under plane-polarized light. **a** Shelly packstone–grainstone (LF9) from Schlattingen, 1154.54. The bioclastic bed is composed of micritized bivalves and significant amounts of intraparticle calcite cement (stained pink) and pore spaces (white). **b** Scoured packstone–grainstone (LF10) from Siblingen, 219.58 m. These beds are composed of micritized sediments, fragmented crinoids, and interparticle cement in white. **c** Drill core image of tempestite (LF10) with scouring and basal crinoidal lag, Schlattingen, 1159.19. **d** Laminated mudstone–wackestone (LF11) composed of marl-rich tempestites with crinoid veneers from Schlattingen, 1166.70 m. **e** Crinoid dominated wackestone–packstone (LF12) composed of large crinoids that are sometimes partially silicified (clear white patch in top right) from Böttstein, 192.97 m. **f** Drill core image of crinoidal bioherm sediments (LF12). Crinoids are extensively bored and the surrounding sediments are filled with fine gastropods and crinoid debris. Schlattingen, 1167.40

*Description*: Lithofacies association 4 occurs near the transition from the Stamberg Member to the Hauptmuschelkalk, i.e. at the change from LFA 2 to LFA 5 (Fig. 3). It is composed of amalgamations of partially dolomitized, cm–dm thick shelly packstones–grainstones (*LF9*). These bioclastic beds are often underlain by heavily bioturbated sediments, and pass upwards into thin mudstones before the next amalgamation of bioclastic facies. Beds show erosive soles, normal grading and horizontal laminations formed by the alignment of fine-grained skeletal particles (Fig. 7a). Bioclastic components are dominated by micritized molluscs and fragmented shells, with minor amounts of brachiopods, glauconite and crinoids.

*Interpretation* Low accommodation space is implied by amalgamated beds, which are unique to this LFA. Packstone–grainstone textures, micritized fragmented shells, good sorting and lack of bioturbation indicate high-energy deposits, but the lack of subsequent bedding features in the overlying mudstones and the short lateral distributions are atypical for tempestites. These facies characteristics are also observed in the massive shelly pack- to grainstones of the Upper Muschelkalk of southern Germany (Aigner 1985; Braun 2003; Koehrer et al. 2010). Facies mapping of shelly pack- to grainstones in southern Germany has shown that these beds were deposited in shallow-water, shore-parallel belts similar to the ooid shoal belts of LFA 3 (Aigner 1985). Based on the similarities between lithofacies association 4 and shelly pack- to grainstones, LFA 4 is interpreted to indicate shelly shoals at the transition from the lagoon to mid-ramp.

### LFA 5 regressive mid-ramp deposits

*Description* Lithofacies association 5 varies from 12 to 20 m in thickness, thus it is the second thickest LFA of the Upper Muschelkalk. It is composed of regularly occurring scoured skeletal wackestone–packstone sheets (*LF10*) (Fig. 7b) and intervening mudstones (*LF3*). Skeletal sheets are characterized by fining-up sequences of molluscs, crinoids, brachiopods gastropods, forams and intraclasts that lay above a scoured sole (Fig. 7c). Large crinoids and lithoclasts often fill the sole. Skeletal sheets at the base of LFA 5 are dominantly mud-supported and crinoid-rich, whereas those at the top are grain-supported and mollusc-rich. Mud contents in individual beds increase upwards and beds often pass upwards into horizontal to angular laminated or hummocky cross-stratified mudstones. Below skeletal sheets, mudstones are often moderately to heavily bioturbated.

*Interpretation* Graded skeletal sheets show all the characteristic textures of tempestites. Normal grading and packstone textures passing upwards into mudstones indicate deposition during waning-energy conditions. Tempestites at the base of LFA 5 show textures and allochems associated with distal tempestites, whereas tempestites at the top indicate deposition in much shallower environments (Aigner 1985). Planar laminations and hummocky cross-stratification associated with tempestites imply that these sediments were deposited below the FWWB. The upwards transitions in tempestite textures indicate that lithofacies association 5 represents a regressive, shallowing-upwards mid-ramp environment. The same tempestite textural evolution is recognized in the Upper Muschelkalk of Germany (Aigner 1985; Braun 2003).

### LFA 6 offshoal nodular mudstones

*Description* This LFA is composed of massive mudstones (*LF3*) and rare wackestone tempestites (*LF9*, *LF11*). The LFA is on average 6 m thick and typically begins within the first 10 m above the Middle Muschelkalk (Fig. 3). The mudstones lack bioturbation except at the top of the LFA where they may be lightly bioturbated. The only sedimentary features are rare, thin horizontal laminations, marly seams encapsulating mudstone nodules and thin crinoid rudstones or mollusc wackestones (*LF11*) that occur near the base of LFA 6 (Fig. 7d).

*Interpretation* The lack of typical tempestite facies, apart from distal tempestites, and the lack of sedimentary features demonstrate that LFA 6 formed below the SWB. Lack of bioturbation may suggest suboxic–anoxic conditions, which are common to the distal ramp environments of many epeiric seas (Tyson and Pearson 1991). The nodular texture has been attributed to burial diagenesis in the German Upper Muschelkalk (Aigner 1985). Accordingly, the massive–nodular mudstones are interpreted as low-energy distal ramp deposits.

### LFA 7 oncoidal tidal channels

*Description* Lithofacies association 7 at the base of the Upper Muschelkalk is composed of a thin (< 2 m) collection of wackestone–packstone oolitic-oncolites (*LF7*) known as the Fützen Bed (Pietsch et al. 2016) or Basaloolith (Merki 1961) within Switzerland and as the Liegend-Oolith in Germany (Paul 1971). The oncolites are composed of non-bioturbated, massive beds of oncoids, which are strongly bored, have shell fragments as nuclei, reach up to 4 cm in thickness and decrease in size upwards. The matrix between the oncoids contains crinoids, sub-centimetre angular black lithoclasts, ooids, anhydrite rosettes and large multi-centimetre mollusc shells. At the top of some beds, both molluscs and anhydrite rosettes are dissolved.

*Interpretation* Well sorted oncoids and the presence of ooids suggest high-energy deposits. Anhydrite rosettes and leached molluscs could indicate that the facies were at times subaerially exposed. The interpretation of this LFA follows that of LFA 3, whereby the ooid-dominated beds are interpreted as shallow oolitic shoals and bars, and oncoidal facies are attributed to tidal channel deposits adjacent to the ooid shoals (Braun 2003; Koehrer et al. 2010).

### LFA 8 transgressive crinoidal deposits

*Description* Transgressive crinoidal deposits are found as an LFA up to 10 m thick at the base of the Upper Muschelkalk. They are composed of crinoid dominated wackestones–packstones (*LF12*), pelitic wackestones (*LF2*), bioturbated mudstones (*LF3*) and rare skeletal sheets (*LF10*). The LFA is best characterized by *LF12*, which shows strongly bioturbated crinoidal and gastropod-rich facies, unique to this LFA (Fig. 7e). Crinoid beds are poorly sorted and contain abundant amounts of bored crinoid ossicles, gastropods and peloids (Fig. 7f). Brachiopods, bivalves and forams are minor constituents of crinoid beds. Non-skeletal beds are strongly bioturbated and sometimes show nodular textures, similar to those of LFA 6. The base of LFA 8 is often a flat-pebble conglomerate, consisting of the subaerially exposed microbial-laminated sheets of the underlying Middle Muschelkalk. The top of the LFA in contrast features mid-ramp tempestite sheets.

*Interpretation* The progression from anhydrite-bearing microbial-laminated Middle Muschelkalk facies to mid-ramp tempestites at the top of the LFA indicate that, unlike other LFA, this association is deepening-upwards. Crinoid packstones at the base of the association are unique to this LFA and are interpreted as lagoon-sheltering crinoidal bioherms that developed during the regressive hemicycle of the Upper Muschelkalk (Aigner 1985). Tempestite facies at the top of the association point to an open-ramp environment at the end of the deposition of LFA 8. Overall, LFA 8 represents transgressive carbonate ramp environments that existed after the drowning of the Middle Muschelkalk evaporites.

### LFA distributions within the revised Schinznach Formation

Prior to this study, the Upper Muschelkalk of the examined boreholes had not been assigned to the revised stratigraphy of the Swiss HARMOS project. Using the new classification criteria of Pietsch et al. (2016), each borehole was divided into the four members of the original Upper Muschelkalk. Each member is composed of one to three lithofacies associations.

The Leutschenberg Member at the base of the Upper Muschelkalk consists of the fully calcitic LFA 6, 7 and 8. Its thickness varies from just > 1 m in Siblingen, where it is composed of only LFA 8, to 15.5 m in Weiach, where all three LFA are present. The base of the Leutschenberg Mb. always corresponds to the top of the Middle Muschelkalk dolomites, whereas the top nearly always corresponds to the top of LFA 6. Therefore, the Leutschenberg Member represents the transgressive, deepening-upwards crinoidal ramp deposits of the Upper Muschelkalk.

The Kienberg Member is primarily composed of LFA 5. Its thickness varies between 11 and 28 m in northern Switzerland. The upper boundary is classified as the last decimetre-scale bed containing > 10 vol% crinoids (Pietsch et al. 2016), which roughly corresponds to the first shoal bodies of LFA 4. However, depending on their crinoid contents, some shoal beds may still form part of the Kienberg Member. The member therefore represents a regressive, shallowing-upwards mid-ramp environment.

The Liedertswil Member is the uppermost member of the Hauptmuschelkalk. Since its upper boundary is marked by the first occurrence of the fully dolomitized Trigonodus Dolomit (Pietsch et al. 2016) and since Upper Muschelkalk dolomitization is discordant to facies (Adams et al. 2019), the Liedertswil Member is not present across Switzerland. In the Lindau well, mid-ramp tempestites with significant crinoid contents are fully dolomitized and therefore the Liedertswil Member is pinched out between the Schlattingen and Benken boreholes (Fig. 3). The Member is composed of parts of LFA 4, 5 and 6, which indicates that it represents the transition from mid-ramp to backshoal environments.

The Stamberg Member corresponds to LFA 1, 2 and 3. Within northern Switzerland, these LFA are always fully dolomitized. Lithofacies association 5 may also be part of the Stamberg Mb., as in areas close to the Vindelician High such as the Lindau well. The thickness of the Stamberg Member is fairly constant between 30 and 40 m on average; however, its thickness decreases westwards from the Vindelician High (Adams et al. 2019). The Stamberg Member represents the sheltered backshoal, peritidal and supratidal environments.

## Sequence stratigraphy

### Third-order sequence

The Upper Muschelkalk reflects a single 3rd-order transgressive–regressive sequence (Aigner 1985; Aigner and Bachmann 1992) as defined by the Transgressive–Regressive Sequence model of Curray (1964) and Embry (1995) and the hierarchical model of Vail et al. (1991). Depending on the placement of the maximum flooding surface (*mfs*), discussed below, the Upper Muschelkalk is either symmetrically or asymmetrically divided, with the regressive hemicycle composing up to three quarters of the Upper Muschelkalk (Aigner and Bachmann 1992; Franz et al. 2015). The 3rd-order cycle began with a transgression over Middle Muschelkalk sulphates and is capped by the transgressive deposits of the Lettenkohle. During each hemicycle, lagoonal, shoal and off-ramp environments were developed. However, the transgressive hemicycle deposits were calcitic and crinoid-dominated, whereas the regressive hemicycle deposits were partially dolomitized and composed of ooid and shelly bioclast shoals (Aigner 1985).

### Maximum flooding surface (mfs)

Locally, the deepest-water facies correspond to the nodular limestones of LFA 6 at the base of the Swiss Upper Muschelkalk. However, the 3rd-order *mfs* does not necessarily correspond to the deepest local facies, since it represents the maximum extent of the entire Upper Muschelkalk Sea across the CEB. The maximum extent of the Upper Muschelkalk Sea has been proposed at various intervals of the Upper Muschelkalk. Kozur (1974) proposed a maximum transgression near the centre of the Upper Muschelkalk, which divided the Upper Muschelkalk into two symmetrical hemi-cycles. Aigner (1985) and Schwarz (1985) refined the maximum transgression to two different “Tonhorizonte” (clay layers) that are regionally correlatable across Germany. Aigner and Bachmann (1992) then attributed the *mfs* to a shale-rich interval in the centre of the Upper Muschelkalk named the “Cycloides-bank”. Franz et al. (2013) recognized that the *mfs* suggested by the previous authors was not characteristic of observations in northern Germany and western regions of the CEB and attributed the *mfs* to a zone of maximum carbonate bed thickness at the base of the Upper Muschelkalk. This zone was later refined to the *Ceratites sequens*/*pulcher* to *philippi*/*robustus* zones (Franz et al. 2015), which would place it well below the *mfs* horizons proposed by Aigner (1985), Schwarz (1985) and Aigner and Bachmann (1992). Unfortunately, a dearth of *Ceratites* and conodonts in the Swiss Upper Muschelkalk precludes any accurate biostratigraphy.

The *mfs* in Switzerland has been attributed to the Dünnlenberg Bed, which corresponds to a high gamma-ray count, marl-rich interval situated at the base of the Liedertswil Member (Pietsch et al. 2016). Ceratites in the Dünnlenberg Bed include C. *compressus, robustus* and *evolutus* (Merki 1961), which would biostratigraphically place it above the *mfs* of Franz et al. (2015). For the purposes of this study, we accept the *mfs* of Pietsch et al. (2016) as an approximate position for the Swiss *mfs* until accurate biostratigraphic analyses can be performed.

### Fourth-order sequences

Stacks of up to five 5th-order sequences compose 5–13 m thick 4th-order cycles (Koehrer et al. 2010; Palermo et al. 2010; Warnecke and Aigner 2019). These cycles have been interpreted as representing sedimentation resulting from long-eccentricity (400-kyr) periods (Warnecke and Aigner 2019) and are regionally correlatable based on biostratigraphic constraints, stratigraphic marker beds, lateral facies correlations, changes in regional facies development, the presence of proximal shoreline outcrops/boreholes and through the multitude of previous regional studies in the southern Germanic basin (Koehrer et al. 2010; Palermo et al. 2010; Warnecke and Aigner 2019). In the case of the northern Swiss Upper Muschelkalk, 4th-order cyclicity is likely present but its reliable identification must await the availability of more information. First, more boreholes are needed. The recognition of Upper Muschelkalk 4th-order cyclicity is an iterative process, whereby cycle boundaries are readjusted upon consideration of each new outcrop and borehole (Palermo et al. 2010). Thus, the nine available boreholes may not be sufficient to accurately represent the 4th-order cyclicity of the entire Swiss Upper Muschelkalk. Second, boreholes closer to the shorelines of the Vindelician High are required. The identification and correlation of 4th-order cycles of the German Upper Muschelkalk was possible due to the correlation of facies from open-marine settings to shoreline proximal settings on the Vindelician High and London Brabant coastlines (Koehrer et al. 2010; Palermo et al. 2010; Warnecke and Aigner 2019b). However, this approach is not yet possible in Switzerland, due to the greater distance of Swiss boreholes from the Vindelician High shorelines (Fig. 1). Furthermore, no biostratigraphic framework exists yet for the Swiss Upper Muschelkalk, which could be used to correlate facies and cycles with respect to the widespread biostratigraphy and cyclicity of the German Upper Muschelkalk.

### Fifth-order sequences

**Fig. 8:**
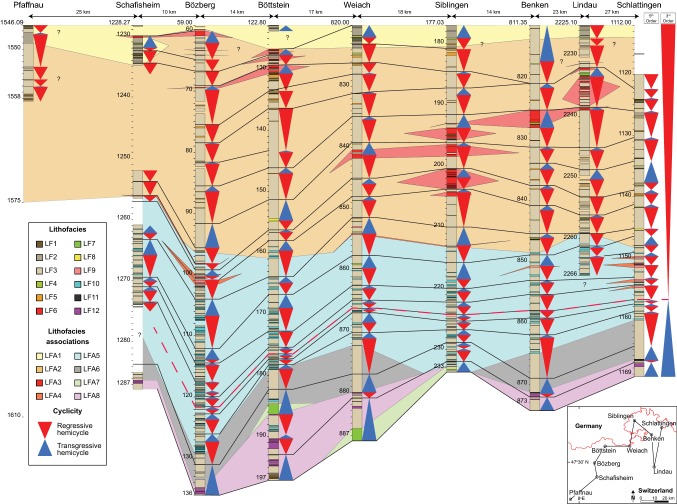
Cyclostratigraphic correlation of borehole data in northern Switzerland. The datum used for correlation is the base of the Keuper. The thick red line corresponds to the maximum flooding surface of the 3rd-order transgressive–regressive sequence of the Upper Muschelkalk, based on the position of the Dünnlenberg bed in Benken after Pietsch et al. (2016). Correlations with Pfaffnau have not been attempted due to the lack of gamma-ray logs, the distance between boreholes and the 53 m of missing drill core

Small-scale cycles with thicknesses of 0.2–7 m are recognized throughout the German Upper Muschelkalk (Aigner 1985; Braun 2003; Koehrer et al. 2010; Warnecke and Aigner 2019). These units have been interpreted as 5th-order cycles, after the hierarchal classification of Vail et al. (1991), and interpreted to reflect the short (100-kyr) orbital eccentricity period (Aigner et al. 1999; Koehrer et al. 2010). We recognized up to 23 small-scale cycles in the 9 studied boreholes and divided them into four cycle types (Fig. 8). Cycles generally begin with a hemicycle consisting of mudstone facies that grade upwards into higher-energy facies, followed by the next hemicycle that shows facies grading upwards back into low-energy mudstone facies. Most cycles correlate laterally across northern Switzerland, however, “missed beats” (Goldhammer et al. 1990) occur due to difficulty in identifying cyclicity in homogenous mudstones and due to erosion of shallow-water facies during regressive phases (Warnecke and Aigner 2019). This particularly affects the top of the Upper Muschelkalk, where an unknown amount of sediment and cycles are missing due to the erosion associated with the Lettenkohle unconformity (Warnecke and Aigner 2019).

#### Backshoal–offshoal cycles

**Fig. 9:**
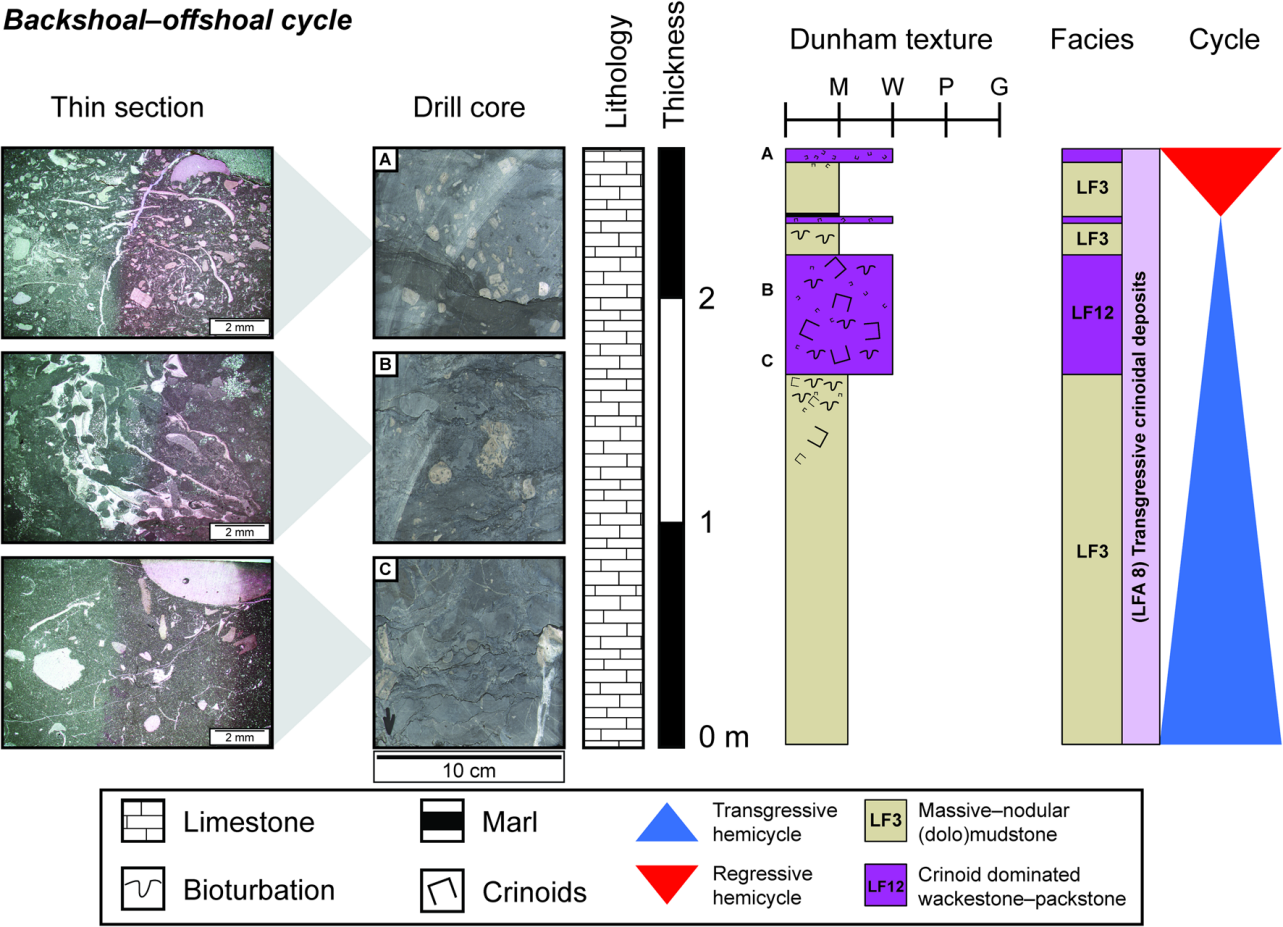
Thin section and drill core photographs, and facies log of one backshoal–offshoal cycle from the base of the Schlattingen borehole. Thin section images taken from locations marked by the letters adjacent to the sedimentary log

These cycles occur during the transgressive 3rd-order hemicycle at the base of the Upper Muschelkalk (Fig. 9). The cycles are asymmetric with the transgressive hemicycle dominating the 2–7 m thickness. In some cases, the regressive hemicycle is not observed. The cycles begin with muddy sediments that transition to crinoid- and gastropod-rich wackestones and packstones (*LF12*), followed by crinoidal wackestone tempestites and mudstones (*LF3*). Distal tempestites (*LF11*) and marls occur in the transgressive hemicycle. These cycles correspond to the crinoidal bank cycles of Aigner (1985).

#### Tempestite cycles

**Fig. 10:**
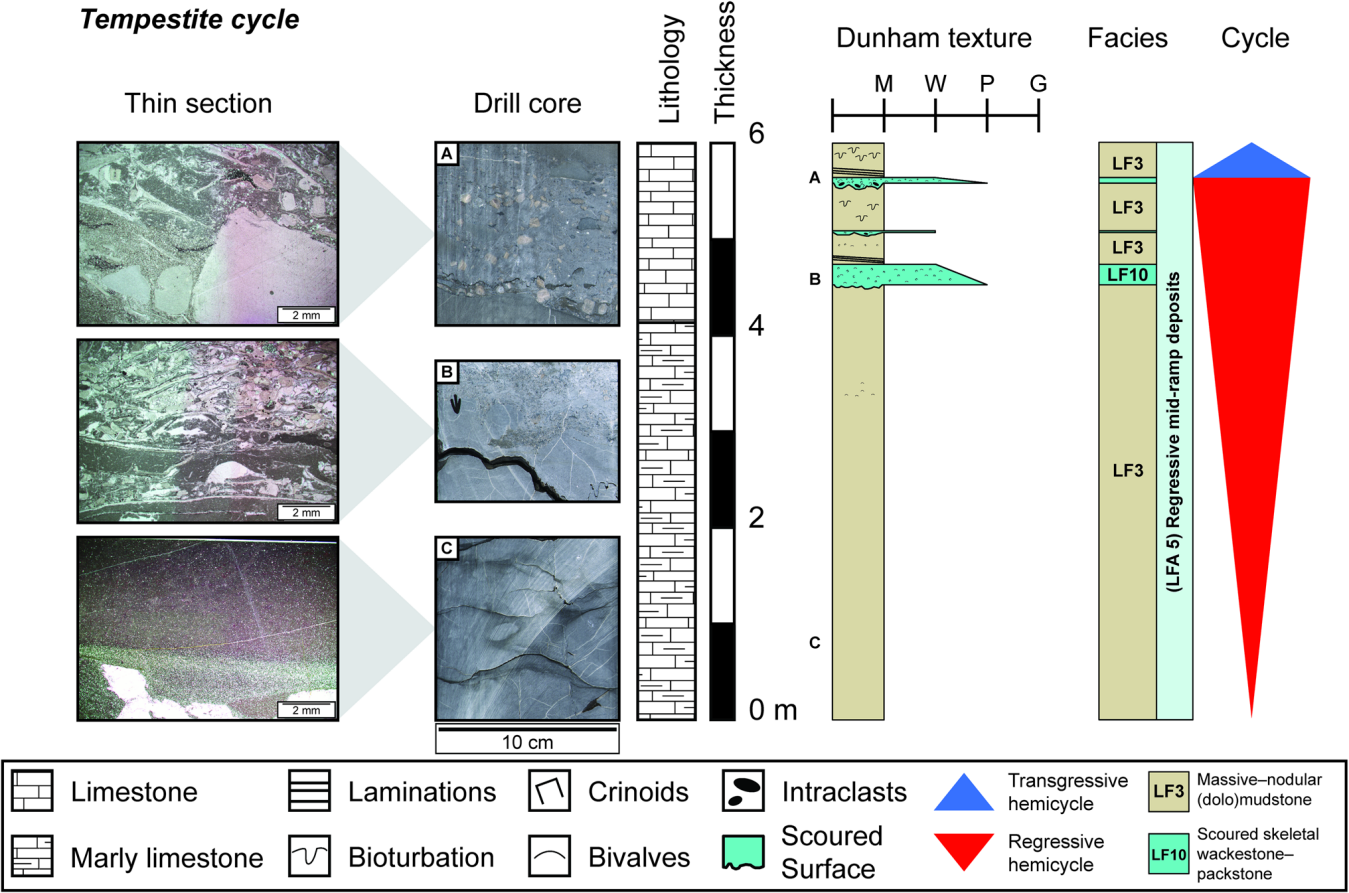
Thin section and drill core photographs, and facies log of one tempestite cycle from the Schlattingen borehole. Thin section images taken from locations marked by the letters adjacent to the sedimentary log

This type of cycle is the most common in the Swiss Upper Muschelkalk and occurs during both hemicycles of the 3rd-order sequence. Tempestite cycles are 2–7 m thick asymmetrical cycles that begin with a thick regressive, shallowing-upwards hemicycle overlain by a thin transgressive hemicycle (Fig. 10). Cycles begin in muddy sediments that pass into a series of coarsening- and thickening-upwards tempestites (*LF10*). Tempestites at the top of the regressive hemicycle contain large crinoid ossicles, intraclasts and large shell debris. During the transgressive hemicycle, tempestite sheets become thinner and finer upwards until they pass into marl-rich, stylolitic mudstones (*LF3*). These cycles correspond to the thickening-upward cycles of Aigner (1985).

**Fig. 11:**
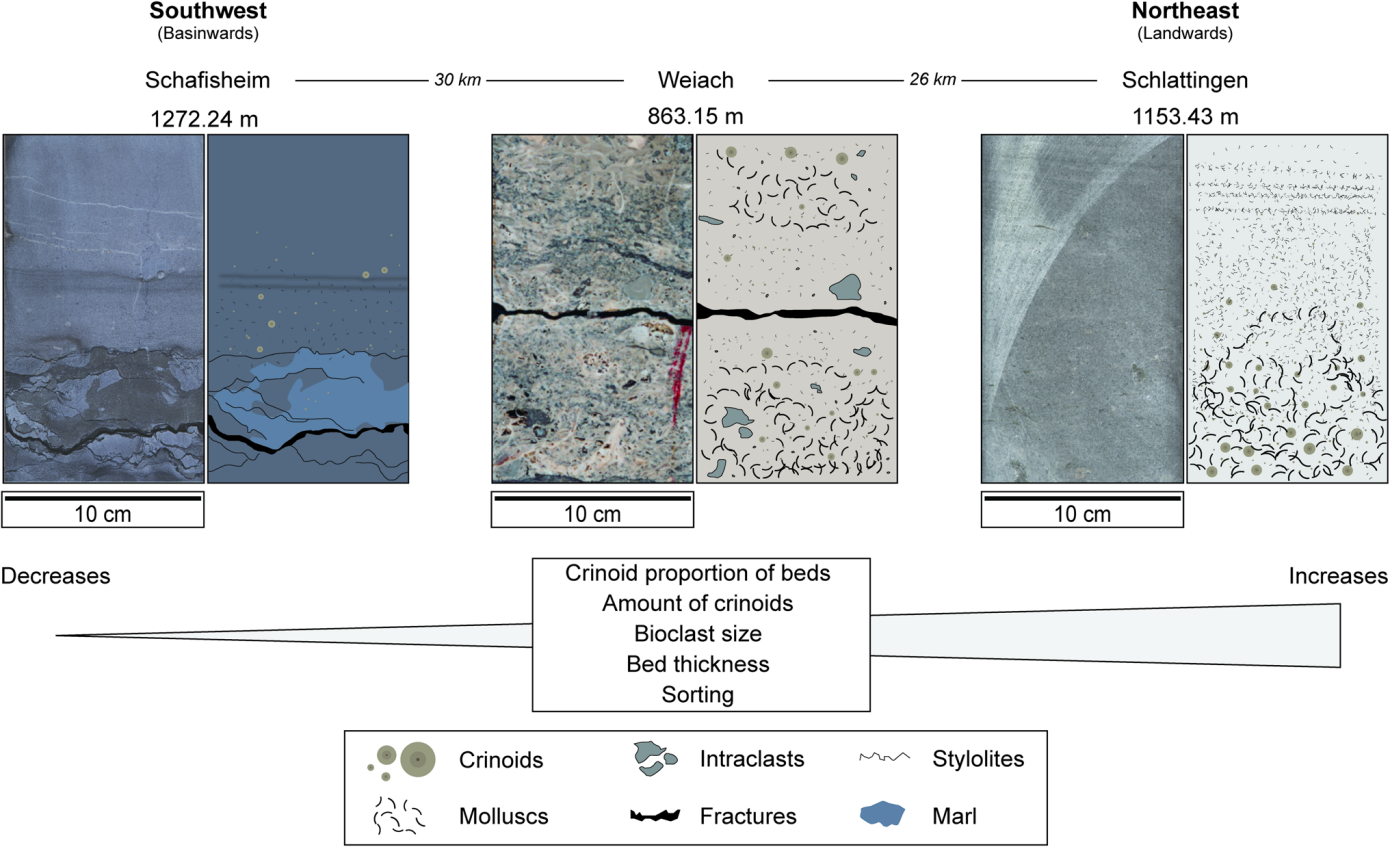
Photographs and explanatory drawings of tempestites from the same tempestite cycle at three boreholes across Switzerland. The illustrations demonstrate the typical textural evolution of tempestites from east to west during the same regressive hemicycle of each 5th-order tempestite cycle

Laterally, tempestites within tempestite cycles record a number of textural and bioclastic changes. As sequences progress westwards into the basin, tempestites within the same sequence show the following changes: scouring decreases, average bioclast size decreases, Dunham textures become muddier, intraclast abundance and sizes decrease, ooid contents decrease, micritization of molluscs decreases and bed thickness decreases (Fig. 11). These trends are observed in all tempestite cycles. A correlation between shoaling facies in the east and proximal–distal tempestites in the west is observed for tempestites at the top of the Hauptmuschelkalk (Fig. 8).

#### Foreshoal- and backshoal cycles

**Fig. 12:**
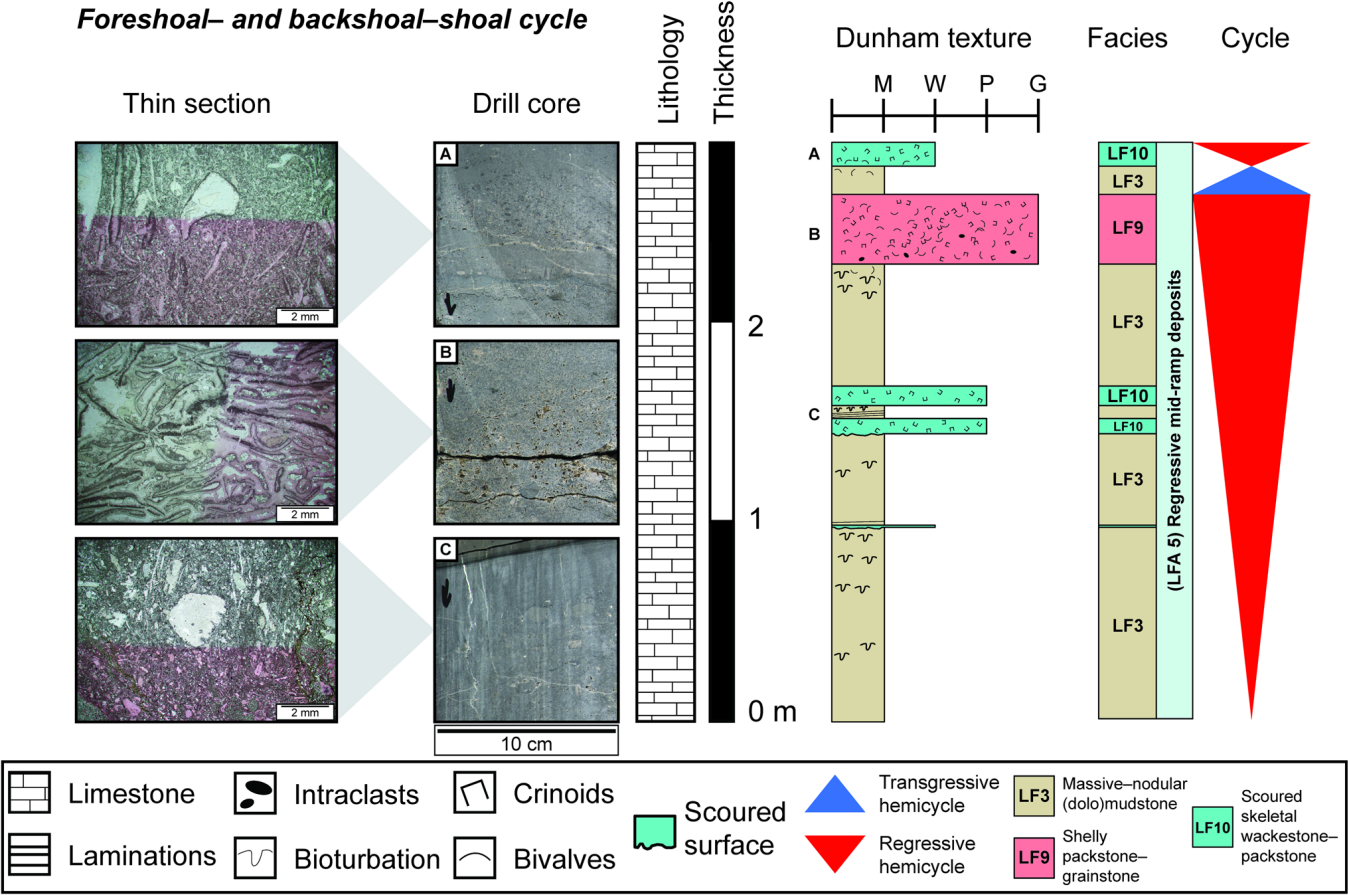
Thin section and drill core photographs, and facies log of one foreshoal–shoal cycle from the Schlattingen borehole. Thin section images taken from locations marked by the letters adjacent to the sedimentary log

These sequences occur in the regressive 3rd-order hemicycle and are ~ 4 m thick symmetrical cycles (Fig. 12). The regressive hemicycle of both cycles begins with mudstones (*LF3*) passing into proximal tempestites (*LF9*) and facies that have been characterised as backshoal washover deposits (*LF5*) in the southern Germanic Basin (Braun 2003; Koehrer et al. 2010; Palermo et al. 2010). Then the deposition of cross-bedded and normally graded shelly and oolitic grainstones (*LF6, 9*) generally marks the end of the regression. In some cases, oncolitic packstones (*LF7*) are considered to be the most regressive facies (Fig. 8; Benken and Lindau). During the transgressive phase of the foreshoal–shoal cycle, mud content increases and Dunham textures wane from grainstones to wackestones. In the backshoal–shoal transgressive hemicycles, oolitic packstones–grainstones (*LF6*) are deposited and depositional energy decreases upwards towards the deposition of wackestone washovers (*LF5*) and massive mudstones (*LF3*). This sequence corresponds to the subtidal shoal cycles of Koehrer et al. (2010) and skeletal bank cycles of Aigner (1985), which are interpreted to represent prograding shoals.

#### Backshoal–peritidal cycles

**Fig. 13:**
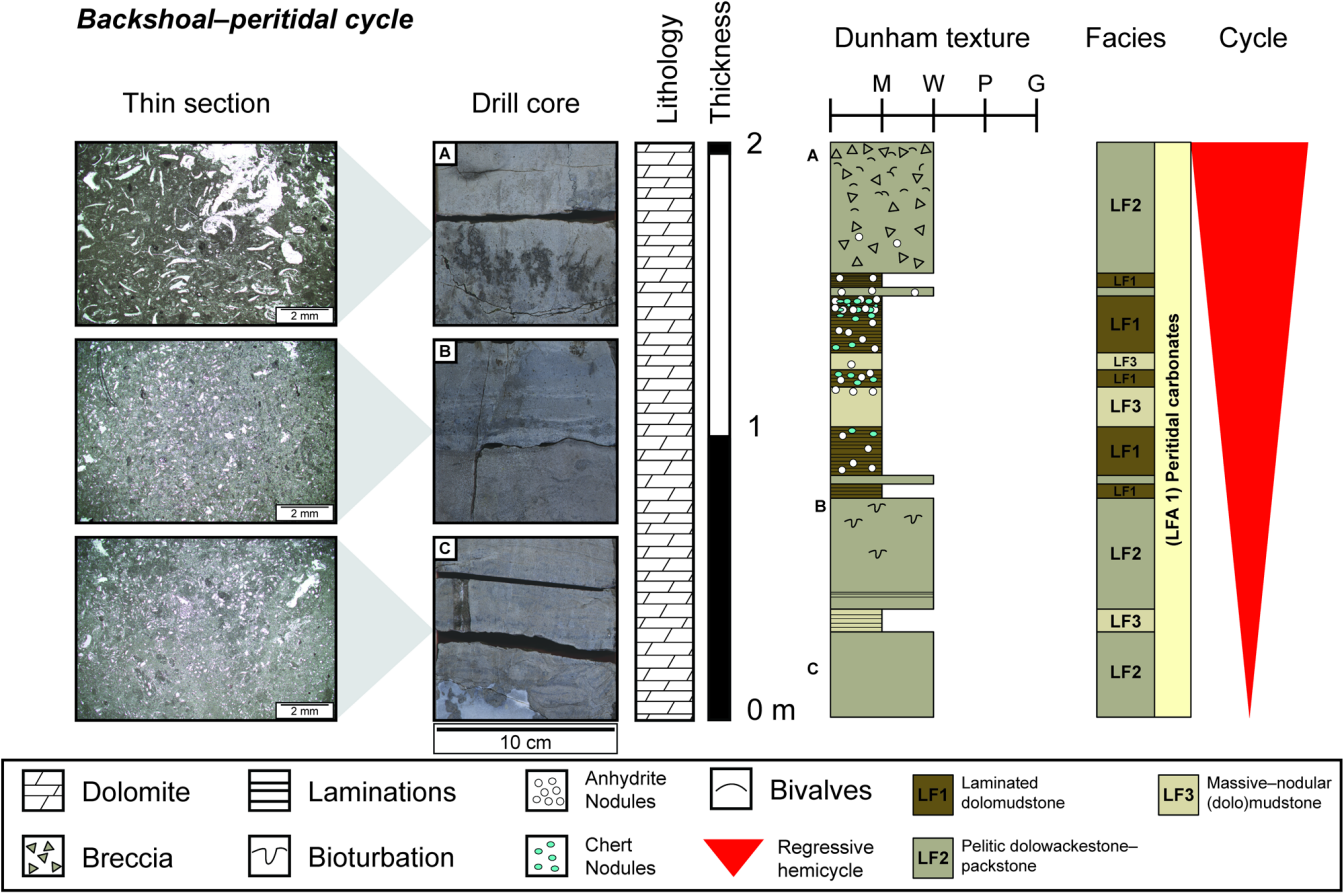
Thin section and drill core photographs, and facies log of one backshoal–peritidal cycle from the top of the Schafisheim borehole. Thin section images taken from locations marked by the letters adjacent to the sedimentary log

These dolomitized regressive asymmetrical cycles occur only at the top of the Trigonodus Dolomit (Fig. 13). Cycle thickness is usually < 3 m. The lower parts of the cycles begin as muddy, bioturbated, peloidal mudstones–wackestones (*LF2*, *3)*, which pass upwards into more strongly bioturbated or massive evaporite-rich peloidal mudstones–packstones (*LF2*) or laminated dolomites (*LF1*). Evaporitic textures increase upwards until reaching a chicken-wire or laminated anhydrite bed that marks the top of the regressive hemicycle. Where the transgressive hemicycle is present, sulphate-rich beds transition over cm–dm to massive/bioturbated mudstones and the cycle then repeats. Backshoal cycles are difficult to correlate between boreholes due the erosional potential of subaerially exposed sediments. These cycles correspond to the backshoal–peritidal cycles of Koehrer et al. (2010).

## Discussion

### Controls on 5th-order Upper Muschelkalk cyclicity

Upper Muschelkalk cyclicity in Germany has been linked to allocyclic tectonic activity and orbitally induced eustasy (Aigner 1985; Aigner et al. 1999; Braun 2003; Koehrer et al. 2010). During the deposition of the Upper Muschelkalk, the southern CEB was a tectonically stable, low thermal-subsidence intracratonic basin, underlain by three Variscan tectonic zones (the Moldanubian, Saxothuringian and Rhenohercynian; Aigner 1985). Increased subsidence of the Saxothuringian during the Middle Triassic led to a thickening of the Upper Muschelkalk in central Germany, which hinders the correlation of 5th-order cycles into the tectonic zone (Aigner 1985). In contrast, the uniform thickness of the Swiss Upper Muschelkalk (Adams et al. 2019), the uniform thickness of the facies sequences (Fig. 8), the absence of any slope break on the Upper Muschelkalk ramp (Aigner 1985; Warnecke and Aigner 2019b) and the location of Switzerland entirely within the Moldanubian zone (Warnecke and Aigner 2019b) rule out any significant tectonic control on Upper Muschelkalk cyclicity within Switzerland.

The Upper Muschelkalk has been estimated to have accumulated in approximately 3.4 Myr (Aigner 1985; Menning et al. 2005). When divided by the 30 cycles observed by Aigner (1985), this results in an average cycle duration of ~ 113 kyr, which corresponds well to the short orbital eccentricity periods of 95– 123 kyr (Berger 1977). If the 23 cycles observed in northern Switzerland correspond to the short orbital eccentricity cycle of ~ 100 kyr, then the Upper Muschelkalk would have formed in roughly 2.3 Myr, which is shorter than the above-mentioned estimates of Aigner (1985) and Menning et al. (2005). However, the difference can be justified by “missed beats” (Goldhammer et al. 1990) and cycles eroded from the top of the Upper Muschelkalk by the Lettenkohle environments. This sedimentary record of orbital eccentricity allows for the regional correlation of cycles and the repeated stacking of carbonate facies sequences. It also suggests that the Burgundy Gate remained partially open until the final stages of Upper Muschelkalk deposition, since ocean-water connections through the Silesian and Moravian Gates had been blocked by the end of the Upper Muschelkalk deposition (Franz et al. 2015).

Sedimentary records of precession and obliquity cycles have not been identified in Upper Muschelkalk sediments. In order to record an orbitally-controlled sea-level change there must have been enough accommodation, a high sedimentation rate and an absence of high-energy erosive events (Strasser 2018). In the case of the Swiss Upper Muschelkalk, the beds most indicative of cycle boundaries are the high energy and erosive tempestites of the open ramp. The frequency of these large-storm events may have inhibited the preservation of precession and obliquity cycles in the sedimentary record. Additionally, most sediments of the Upper Muschelkalk are homogeneous mudstones and cycle boundaries and changes in water depths resulting from short (< 100 kyr) orbitally induced sea-level changes cannot be inferred from these mudstones.

### Spatial evolution of tempestites

Mesozoic storm-dominated ramps are replete with tempestites whose textures, components, thicknesses and abundances indicate local depositional settings and palaeo-water depths (Aigner 1985; Immenhauser 2009; Pérez-López and Pérez-Valera 2012). By examining the vertical and lateral progressions of tempestite textures within tempestite cycles, cycles can be characterised as shallowing or deepening upward (Immenhauser 2009). Lateral facies transitions can furthermore indicate the position and source of tempestites relative to shoals and palaeoshorelines (Pérez-López and Pérez-Valera 2012). Vertical successions of tempestites in the Swiss Upper Muschelkalk indicate that the lower hemicycle of each individual tempestite cycle was shallowing upwards and deposited in a progressively shallower environment; however, tempestites can be deposited in both shallowing-upwards backshoal and foreshoal environments. Tempestites derived from shoal-proximal environments should contain components characteristic of shoals, and those sourced from palaeoshorelines should represent peritidal sediments.

Peritidal sediments from the top and bottom of the Upper Muschelkalk are peloidal, crinoid-poor, mollusc-rich and mud-rich (Aigner 1985; Braun 2003; Koehrer et al. 2010). If tempestites were produced from peritidal environments, they would be characterized by peloidal, crinoid-lacking and mollusc-rich sediments. Such facies are observed in the Trigonodus Dolomit, i.e. *LF2, 4, 5, 8*, but they do not characterize the tempestite facies (*LF10)* of the Hauptmuschelkalk. The mollusc- and crinoid-rich, peloid-lacking and micritized components of Swiss tempestites are analogous to the components of oolitic and shelly shoals, and crinoid bioherms. Furthermore, lateral correlations of tempestites with shoals demonstrate that these tempestites were sourced from shoals in the area of the eastern boreholes (Fig. 8). Tempestites in southern Germany are correlatable over tens of km and demonstrate that they too were sourced from shoal-proximal environments (Aigner 1985; Koehrer et al. 2010; Palermo et al. 2010).

In the gradient-current model of Aigner (1985), tempestites record the effects of decreasing depositional energy and increasing water depth as they are deposited away from their component source. As tempestites are deposited, the waning depositional energy and the corresponding increase in water depth are indicated by decreasing amounts of intraclasts, decreasing intraclast sizes, decreasing grain/bioclast sizes, increased mud contents, textural changes from packstones–mudstones and reduced scouring. Proximal tempestites show ripple laminations, parallel laminations and bed amalgamation, whereas distal tempestites may show no features at all, or only marly beds following thin tempestite deposits (Aigner 1985). All these features indicating decreasing energy conditions are observed when Swiss Hauptmuschelkalk tempestite cycles are traced from eastern to western Switzerland (Fig. 11). This demonstrates that the Swiss tempestites were sourced from shoal bodies in eastern Switzerland and deposited in progressively deeper environments basinward into western Switzerland. Shoals progressively prograded across the basin during the 3rd-order regressive hemicycle and with them, so did tempestites (Fig. 8). This shows that Hauptmuschelkalk tempestites were dominantly formed on the foreshoal ramp of the Upper Muschelkalk and not within the backshoal lagoon. Backshoal lagoonal sediments do contain washover deposits and rare tempestites (Fig. 8; Böttstein); however, their characteristics, components and textures differ considerably from the tempestites of the Hauptmuschelkalk (Table 1).

Despite the lateral depositional extent of tempestite sheets, it is unlikely that tempestites in different boreholes record the same storm event. Major hurricane-sized storms, like those responsible for Upper Muschelkalk tempestites, occur once every 400–18 000 years (Molina et al. 1997). During a cycle of 100 kyr, dozens of major storm events would occur, despite the few preserved tempestites. Outcrop studies of the Upper Muschelkalk that have laterally traced tempestite sheets over kilometres also show a high variability in the number of tempestites within a single correlatable cycle (Palermo et al. 2010). Without closer borehole spacing, the lateral continuity of individual tempestites is impossible to verify.

### Upper Muschelkalk ramp evolution in Switzerland

The facies of the Swiss Upper Muschelkalk indicate deposition on a homoclinal carbonate ramp. Upwards and lateral facies transitions and tempestite characteristics (Aigner 1985) permit the recognition of temporal and spatial changes in depositional energy and depositional settings during the formation of the Upper Muschelkalk ramp. The evolution of the ramp is divided into two distinct phases corresponding to the transgressive and regressive hemicycles of a 3rd-order sequence. During the transgressive hemicycle, depositional energy decreased upwards from backshoal to off-ramp facies. In the regressive hemicycle the depositional energy peaked on mid-ramp to shoal facies, where the impact of storm activity was greatest, then decreased upwards to backshoal and peritidal sediments.

#### The transgressive crinoidal ramp

**Fig. 14:**
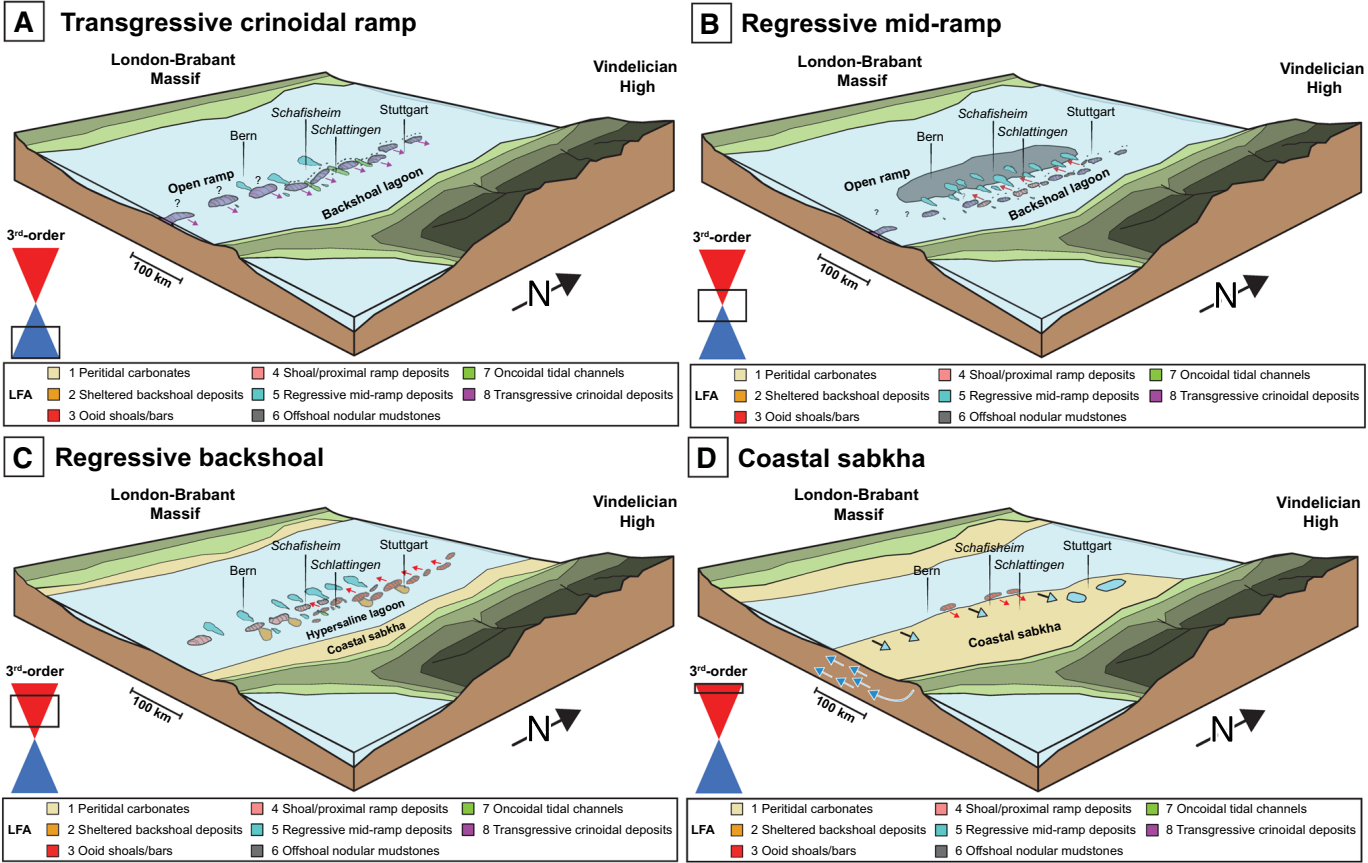
Palaeogeographic evolution of the southern Central European Basin (CEB) during the deposition of the Upper Muschelkalk. Modified from Adams and Diamond (2017). The box around the transgressive–regressive (T–R) hemicycles refers to the time interval shown in the corresponding figure, in relation to the third-order T–R cycle of the Upper Muschelkalk deposition. **a** During the transgressive 3rd-order hemicycle, crinoid shoals and small bioherms develop and retrograde (denoted by arrows) towards the Vindelician High. Some tempestites are deposited on the open ramp and in the backshoal lagoon. **b** Shelly shoals develop in eastern Switzerland and begin to prograde (denoted by arrows). Nodular mudstones are deposited on the open ramp of the southern CEB and are subsequently covered by tempestite sheets during the regressive 3rd-order hemicycle. **c** Oolitic shoals develop in the backshoal and prograde westwards (denoted by arrows). Backshoal washover deposits begin to cover the now hypersaline backshoal lagoon. A coastal sabkha develops on the shoreline of the Vindelician High. **d** After the coastal sabkha has prograded far into the basin, it is subsequently overlain by retrograding oolitic shoals (denoted by red arrows) in northern Switzerland during a late marine transgression (light blue arrows). Meteoric groundwaters are seen percolating into the basin (dark blue arrows; Adams and Diamond; 2017)

The Middle–Upper Muschelkalk transition begins with a distinct facies and palaeoenvironmental modification of the southern CEB. Upon the opening of the Burgundy Gate, Tethyian seawaters transgressed the southern CEB and Middle Muschelkalk dolo-laminates and evaporites were abruptly overlain by carbonate muds. Tethyian crinoids rapidly colonized the southern CEB and established bioherms and banks across northern Switzerland (Fig. 14a). Subsequently, the basin experienced the 3rd-order maximum flooding interval while the Upper Muschelkalk in Switzerland developed into a storm-dominated homoclinal carbonate ramp.

Homoclinal carbonate ramps develop in low tectonic-activity settings such as landward from continental margins, on foreland basins or in continental interiors (Read 1985). Middle Muschelkalk facies demonstrate that the southern CEB had a planar basin morphology prior to Upper Muschelkalk basin subsidence (Geyer and Gwinner 2011). In order to maintain the homoclinal nature of the carbonate ramp during the transgression, sedimentation and subsidence rates must have remained practically equal across northern Switzerland. Lateral cycle correlations and equal sediment thicknesses during the transgressive 3rd-order hemicycle support this hypothesis (Fig. 8). Although framework-producing biota, such as the crinoidal bioherms, can modify ramp morphologies (Pomar 2001), their presence in northern Switzerland did not have an appreciable effect on the sedimentation of the Upper Muschelkalk ramp. This may be due to the patch-reef-like distribution of Upper Muschelkalk crinoid bioherms (Hagdorn 2006; Diedrich 2017) or potentially to the lack of high-energy crinoid banks in Switzerland. Bioherm thicknesses do not exceed two meters and despite shallow water depths, bioherms were likely never large enough to restrict and constrain sediments to the backshoal environments. Other frame-building organisms such as corals or sponges are rarely observed in the southern CEB but were important constituents in the Spanish and Polish Upper Muschelkalk (Calvet and Tucker 1995; Tucker and Marshall 2004; Matysik 2016).

Crinoid bioherms during the transgressive hemicycle were characterized by sediments filled with numerous cm-sized bored crinoid ossicles, peloids and mm-sized gastropods (Fig. 9). Some gastropod species have been known to have had parasitic relationships with crinoids (Bandel 1992), which could explain the extensive boring of crinoids and peloidal content of crinoid bioherms. Crinoid tempestites deposited adjacent to the crinoidal mounds also contain the large bored crinoids that characterize the crinoidal mound facies, which suggests that the crinoid ossicles were sourced and transported from these mounds. Since crinoids are stenohaline organisms, their presence as bioherms suggests that the Upper Muschelkalk Sea during the transgressive ramp had normal salinities and temperatures, unlike the arid hypersaline conditions encountered during the regressive 3rd-order hemicycle (Schauer and Aigner 1997).

During the second 5th-order transgressive cycle at the base of the Upper Muschelkalk, up to 7 m of massive, nodular mudstones were deposited (LFA 6). Intervals of nodular mudstones had been interpreted in Germany as features resulting from the pressure solution of layered shalely limestones in quiet backshoal or basinal environments (Aigner 1985). The nodular mudstones of northern Switzerland can be attributed to a low-energy fair-weather wave-base (FWWB) to storm wave-base (SWB) zone of a backshoal or below the SWB in a basinal environment based on their massive texture, rare tempestites, moderate bioturbation and high clay contents. However, massive mudstone formation is unlikely to have occurred in the backshoal environment for a number of reasons. If seven metres of mudstones could be deposited above the *SWB* without any intercalating tempestites it would imply either rapid deposition, which is unlikely based on the large thickness of mud, or deposition during a period of low storm activity. Massive mudstone formation during a period of low storm activity is also unlikely, because distal tempestites are still observed at the base of the nodular mudstones and within the sequence as thin marl-rich crinoidal rudites. As discussed above, early crinoidal banks in Switzerland were not large enough to baffle the effects of strong storms, and the presence of tempestites within backshoal environments (Fig. 8; Böttstein) indicates that storm events were indeed capable of overcoming crinoid banks. Nodular mudstones, therefore, most likely did not form in a sheltered, low-energy backshoal environment. Only a basinal environment would be consistent with the presence of distal tempestites, lack of any other intercalated tempestites, abundant clay contents, and the position on the deepening-upward transgressive ramp.

Following the deposition of nodular mudstones, three to five shallowing-upwards tempestite cycles were deposited on the open ramp. These initial tempestite cycles are identical to the tempestite cycles of the regressive ramp, apart from higher crinoid contents at the tops of their regressive hemicycles.

#### The regressive oolitic ramp

The transition from the transgressive to the regressive ramp is not distinguished by any specific facies or depositional environment and without the use of gamma-ray logs, the *mfs* cannot be placed accurately in any individual facies within the studied cores. The transition begins with the continuation of shallowing-upwards tempestite cycles. Tempestites at cycle tops begin to show micritized components, they become more mollusc-dominated and they contain fewer crinoids as cycles progress upwards. These changes indicate depositional environments that were progressively approaching shoals during the overall ramp regression (Braun 2003; Ruf and Aigner 2004). After three to seven tempestite cycles, shelly shoals had developed across most of northern Switzerland.

Shelly shoals initially developed in north-eastern Switzerland and then prograded westwards with each cycle (Fig. 14b). These shoals were thin (< 1 m thickness) and surrounded by muddy sediments, unlike the multi-metre thick, mud-free shelly/oolitic shoals of southern Germany (Braun 2003; Kostic and Aigner 2004; Ruf and Aigner 2004). As the lateral continuation of shoals is on the order of kilometres (Koehrer et al. 2010; Palermo et al. 2010), Swiss shoal facies and washovers of the same cycle may represent continuous shoaling bodies between boreholes (Fig. 8). In Germany, Upper Muschelkalk shoals mark the transition from the open ramp to the sheltered backshoal lagoon (Aigner 1985; Koehrer et al. 2010; Palermo et al. 2010). Despite the small thicknesses of Swiss shoals, the low-energy facies eastwards and above shelly shoals indicate sheltered depositional environments, unlike the environments that preceded the deposition of shelly shoals.

Following shelly shoal formation, the ramp experienced one to two cycles of muddy sedimentation with only the occasional interruption of washover deposits. The backshoal environment at this time was oxygenated and of normal salinity as demonstrated by the strongly bioturbated and evaporite-free sediments, unlike the backshoal sediments at the top of the Trigonodus Dolomit. Backshoal sediments are always dolomitized within northern Switzerland; however, dolomitization occurred much later in the depositional history of the Trigonodus Dolomit and was unrelated to these initial backshoal sediments (Adams et al. 2019).

During the evolution of the backshoal environment, low-energy backshoal deposits were interrupted by high-energy, multi-metre thick, mud-poor, cross-bedded oolitic shoals (LFA 3) (Fig. 14c). Oolitic shoals of the Upper Muschelkalk of southern Germany are up to 30 km in length and 15 km in width and developed as progradational bodies during regressive sequences (Palermo et al. 2010). They occur in the same locations as the underlying crinoidal banks of the transgressive ramp and were influenced by the subtle relief created by crinoidal banks or by minor palaeohighs that rimmed the shorelines of the Vindelician High (Aigner 1985; Braun 2003; Geyer and Gwinner 2011; Warnecke and Aigner 2019). However, palaeotectonic maps of northern Switzerland indicate that no NNE–SSW trending palaeohighs are present in Northern Switzerland (Madritsch 2015). Instead, northern Switzerland is underlain by a large WSW–ENE trending permocarboniferous trough (PCT) and numerous WNW–ESE trending fault structures (Thury et al. 1994). These maps indicate that Swiss oolitic shoals are present in areas within and outside the PCT (Marchant et al. 2005) and that therefore they bear no relation to the underlying palaeotectonic structures. Of the Swiss Upper Muschelkalk oolites, only the oolitic shoal near Canton Schaffhausen is similar to the German shoals (Fig. 4; Siblingen). Other oolitic shoals are on average < 10 km in width, they are developed across northern Switzerland and they have no association with crinoid banks or shelly shoals. Therefore, the controls on the locations of the Swiss oolites remain unknown.

After oolite development, backshoal sediments became increasingly bioturbated, peloidal, and anhydrite-rich. Storm washovers continued to be deposited but their frequency waned as lagoonal facies passed upwards into shoreline proximal environments. Washover compositions, exclusive of crinoids and brachiopods, which were formerly present in backshoal sediments, may point to increased salinities in the backshoal lagoon during the later cycles (Palermo et al. 2010). Additionally, cycle tops begin to be capped by nodular evaporites across the basin (Fig. 13). Following evaporite formation, the overlying sediments are typically evaporite-poor, bioturbated sediments, which indicates a return to normal salinity conditions and sedimentation with each new cycle, prior to the next formation of evaporites.

Sediments of the last few cycles of the Upper Muschelkalk, which were not eroded away at the top of unit, reflect the increasing restriction and sea-level fall of the Upper Muschelkalk Sea. These sediments, replete with palaeosol, microbial laminates, brecciation, shrinkage cracks and extensive evaporite deposits and their dissolution vugs, have been found from the Netherlands to southern Germany along the palaeoshorelines of the Vindelician High and Rhenish Massif (Schauer and Aigner 1997; Braun 2003; Pöppelreiter et al. 2003; Koehrer et al. 2010; Warnecke and Aigner 2019). In Switzerland, however, the thickest Upper Muschelkalk evaporites were deposited in the central parts of the basin, in locations > 100 km seaward from the Anisian/Ladinian palaeoshorelines, as seen in the boreholes of Pfaffnau and Schafisheim (Fig. 3) and Courtion (Fischer and Luterbacher 1963).

The chicken-wire anhydrites, anhydrite nodules and lenticular anhydrite laths of northern Switzerland can reasonably be attributed to a coastal sabkha environment. Deep-water conditions during evaporite formation can be ruled out in the Upper Muschelkalk Sea due to the shallowness of the epeiric sea, the surrounding intertidal microbial-laminates and lack of turbidite mass flows. Continental settings are also unlikely based on the Middle Triassic seawater ^87^Sr/^86^Sr ratios of the primary anhydrites, which reflect only minor amounts of strontium derived from continental runoff (Adams et al. 2019). A shallow subaqueous origin for the Swiss evaporites is furthermore unlikely since evaporites deposited from subaqueous settings are typically m–dm thick bodies, show increasing variability of biota upwards, show rhythmic bedded evaporite–carbonate units, have low dolomite contents and do not show subaerial exposure indicators such as enterolithic and nodular anhydrites, microbial laminates and desiccation cracks (Davies and Nassichuk 1975; Warren 2006). In contrast, the studied evaporites are thin (< 1 m thick), show no rhythmic bedding, their variability of biota decreases upwards, they are rich in dolomite, they contain subaerial exposure features and they display nodular and enterolithic anhydrites. These evaporite sediments demonstrate that, at the end of the deposition of the Upper Muschelkalk, all of northern Switzerland was covered by a large coastal sabkha extending over 150 km into the basin from the western palaeoshorelines of the Vindelician High (Fig. 14d). The lack of any significant evaporite deposits in eastern Switzerland is explained by the erosional loss of meters of rock at the angular unconformity between the Upper Muschelkalk and the overlying Lettenkohle (Merki 1961). Diagenetic anhydrite nodules throughout eastern Switzerland indicate the presence of hypersaline environments supersaturated with respect to anhydrite, despite the apparent lack of hypersaline facies at the conclusion of Upper Muschelkalk deposition (Adams et al. 2019).

Prior to the erosion at the top of the Upper Muschelkalk in northern Switzerland, one final oolitic structure was deposited during the one to two cycles above the sabkha facies. These oolites, the “Kaistener Schichten” after Merki (1961), are laterally continuous in a W–E trend over several kilometres (Fig. 3). Using the Persian Gulf as a palaeogeographic proxy, these oolites could have formed in the deep basin, in tidal channels, on beaches, in the open lagoons or in desert environments (Loreau and Purser 1973). Beach and desert/aeolian depositional environments of the Upper Muschelkalk and the Persian Gulf both show the presence of detrital quartz grains between ooids and as ooid nuclei (Loreau and Purser 1973; Braun 2003), whereas the Kaistener Schichten microfacies contain no detrital quartz (Fig. 5d). Additionally, the oolites lack mud and are well sorted, which suggest deposition in a high-energy environment not present in deeper basinal settings nor in a quiet sheltered lagoon. These observations suggest that the Kaistener Schichten represent the development of a large shoal in central Switzerland and southern Germany at the end of the Upper Muschelkalk deposition. Considering the underlying supratidal facies and basin morphology, the shoal must have retrograded eastwards towards the Vindelician High during a seawater transgression. As the facies overlying the Kaistener Schichten have been eroded, the final environments of the Upper Muschelkalk are unknown; however, microfacies of the Kaistener Schichten show anhydrite cementation, which indicates the formation of an environment capable of producing anhydrite-supersaturated and dolomitizing brines following the deposition of the oolite shoal, in agreement with Adams et al. (2019).

The results and interpretations presented herein demonstrate a variable ramp evolution with a wide variety of environments and depositional energies within the Swiss Upper Muschelkalk. Palaeogeographic maps of the southern CEB during the Middle Triassic have all suggested that Switzerland represented an extensive backshoal environment during the entire deposition of the Upper Muschelkalk (Alesi 1984; Aigner 1985; Ziegler 1990; Franz et al. 2015). Whereas backshoal environments were indeed widespread in Switzerland during the deposition of the Trigonodus Dolomit and the lowermost few metres of the Hauptmuschelkalk, in this study we have found that up to nearly half of the Upper Muschelkalk deposition occurred on an open ramp. Because of this, porous shoals and marginal shoal facies were only minor constituents of the Swiss Upper Muschelkalk, unlike in the German sediments. This paucity of porous shoals and marginal facies, along with the effects of early marine, mixing-zone and meteoric cementation (Adams and Diamond 2017) and burial compaction and late dolomite cementation (Aschwanden et al. 2019), resulted in the generally poor primary reservoir properties of the Upper Muschelkalk of northern Switzerland.

## Conclusions

The Middle Triassic Upper Muschelkalk of Switzerland constitutes the carbonate deposits of a 3rd-order transgressive–regressive sequence on a storm-dominated homoclinal carbonate ramp in the semi-enclosed southern Central European Basin (CEB). The Upper Muschelkalk was deposited in a shallow epeiric sea that was separated from the Tethys Ocean by three tectonically controlled gates that periodically allowed and restricted the flow of Tethyian waters into the basin. The basin’s restriction, along with the shallowness of the epeiric sea, made the Upper Muschelkalk particularly sensitive to even minor sea level fluctuations.

Periodic Tethyian transgressions, driven by orbital eccentricity-induced sea-level fluctuations, led to the deposition of at least 23 m-scale 5th-order cycles in the Swiss Upper Muschelkalk. Unlike the 5th-order cycles of the Upper Muschelkalk of southern Germany, Swiss cyclicity was not affected by local tectonism. Lateral correlation of 5th-order cycles demonstrates that, during the initial 3rd-order transgressive hemicycle, crinoid bioherms developed across the southern CEB and were subsequently buried by deeper-water distal-ramp sediments. This contrasts with prior palaeogeographic reconstructions of Switzerland as a backshoal environment. Following the distal ramp sedimentation, a series of shallowing-upwards tempestite sequences were deposited prior to and after the maximum flooding surface of the basin-wide 3rd-order sequence. Lateral correlations of tempestite cycles demonstrate the progressive tempestite evolution across the basin as a result of deepening water and loss of depositional energy.

During the regressive 3rd-order hemicycle, tempestite sequences continued to be deposited until the formation of shelly shoals. Shelly shoals prograded westwards across Switzerland over a period of ~ 300 kyr, such that they can now be found in north-central Switzerland. The shoals separated the open ramp from the sheltered backshoal and induced a sharp facies boundary between the high-energy, well-sorted tempestites stratigraphically below, and the backshoal muddy sediments stratigraphically above the shelly shoals. Backshoal sedimentation was only interrupted by the development of ooid shoals prior to the formation of coastal environments.

At the end of the deposition of the Upper Muschelkalk in Switzerland, a coastal sabkha prograded across the country. In eastern Switzerland, evidence for the sabkha was eroded by the Lettenkohle environment but thick evaporites are increasingly common westwards into the basin. Following the development of the coastal sabkha a major transgression deposited a retrogradational oolitic shoal in northern Switzerland. This final transgression preceded the development of the hypersaline environment that produced the dolomitizing brines of the Trigonodus Dolomit.

Varied depositional environments and a varied ramp evolution in the southern CEB led to differences in the reservoir properties of the facies of the Swiss and southern German Upper Muschelkalk. Open ramp conditions were more predominant during the deposition of the Upper Muschelkalk in Switzerland than in Germany, which led to the deposition of numerous low reservoir-quality tempestites. This contrasts with the numerous oolitic- and shelly-shoal facies of the backshoal sediments of the German Upper Muschelkalk along the shorelines of the Vindelician High. A lack of porous shoals, along with the effects of early diagenesis and burial compaction resulted in the poor reservoir properties of the Upper Muschelkalk of Switzerland.

## References

[CR1] Adams A, Diamond LW (2017). Early diagenesis driven by widespread meteoric infiltration of a Central European carbonate ramp: A reinterpretation of the Upper Muschelkalk. Sedimentary Geology.

[CR2] Adams A, Diamond LW, Aschwanden L (2019). Dolomitization by hypersaline reflux into dense groundwaters as revealed by vertical trends in Sr- and O-isotopes: Upper Muschelkalk, Switzerland. Sedimentology.

[CR3] Aigner T (1985). Storm depositional systems. Dynamic stratigraphy in modern and ancient shallow marine sequences.

[CR4] Aigner T, Bachmann GH (1992). Sequence-stratigraphic framework of the German Triassic. Sedimentary Geology.

[CR5] Aigner T, Hornung J, Junghans W-D, Pöppelreiter M (1999). Baselevel cycles in the Triassic of the South-German Basin: A short progress report. Zentralblatt für Geologie und Paläontologie Teil.

[CR6] Alesi, J. E. (1984). Der Trigonodus-Dolomit im Oberen Muschelkalk von SW-Deutschland. In *Arbeiten aus dem Institut für Geologie und Paläontologie an der Universität Stuttgart* (Vol. 79, pp. 1–53).

[CR7] Aschwanden L, Diamond LW, Adams A (2019). Effects of progressive burial on matrix porosity and permeability of dolostones in the foreland basin of the Alpine Orogen, Switzerland. Marine and Petroleum Geology.

[CR8] Bandel K (1992). Platyceratidae from the Triassic St. Cassian Formation and the evolutionary history of the Neritomorpha (Gastropoda). Paläontologische Zeitschrift.

[CR9] Berger A (1977). Support for the astronomical theory of climate change. Nature.

[CR10] Borkhataria RA, Aigner T, Pöppelreiter MC, Pipping JC (2005). Characterisation of epeiric “layer-cake” carbonate reservoirs: Upper Muschelkalk (Middle Triassic), the Netherlands. Journal of Petroleum Geology.

[CR11] Braun, S. (2003). Quantitative analysis of carbonate sandbodies: Outcrop analog study from an epicontinental basin (Triassic, Germany). Doctoral dissertation. University of Tübingen, Tübingen, Germany.

[CR12] Burchette TP, Wright VP (1992). Carbonate ramp depositional systems. Sedimentary Geology.

[CR13] Calvet F, Tucker ME, Monty C, Bosence D, Bridges P, Pratt B (1995). Mud-mounds with reefal caps in the upper Muschelkalk (Triassic), eastern Spain. Mud mounds: Origin and evolution. IAS, special publications.

[CR14] Chevalier G, Diamond LW, Leu W (2010). Potential for deep geological sequestration of CO_2_ in Switzerland: A first appraisal. Swiss Journal of Geosciences.

[CR15] Curray JR, Miller RL (1964). Transgressions and regressions. Papers in marine geology. Sheppard commemorative volume.

[CR16] Davies GR, Logan BW, Davies GR, Read JF, Cebulski DE (1970). Algal-laminated sediments, Gladstone Embayment, Shark Bay, Western Australia. Carbonate sedimentation and environments, Shark Bay, Western Australia.

[CR17] Davies GR, Nassichuk WW (1975). Subaqueous evaporites of the Carboniferous Otto Fiord Formation, Canadian Arctic Archipelago: A summary. Geology.

[CR18] Deutsche Stratigraphische Kommission (2002). Stratigraphische Tabelle von Deutschland 2002.

[CR19] Dickson JAD (1966). Carbonate identification and genesis as revealed by staining. Journal of Sedimentary Petrology.

[CR20] Diedrich C (2017). Tsunami killed and backwashed accumulated crinoids in Middle Triassic (Anisian) intracratonic Germanic Basin carbonates of central Europe. Carbonates and Evaporites.

[CR21] Disler C (1914). Stratigraphischer Führer durch die geologischen Formationen im Gebiet zwischen Aare, Birs und Rhein.

[CR22] Dunham RJ, Ham WE (1962). Classification of carbonate rocks according to depositional texture. Classification of carbonate rocks: A symposium.

[CR23] Embry A (1995). Sequence boundaries and sequence hierarchies: Problems and proposals. Norwegian Petroleum Society Special Publications.

[CR24] Fischer H, Luterbacher H (1963). Das Mesozoikum der Bohrungen Courtion 1 (Kt. Fribourg) und Altishofen 1 (Kt. Luzern). Beiträge zur Geologischen Karte der Schweiz, Neue Folge.

[CR25] Franz M, Henniger M, Barnasch J (2013). The strong diachronous Muschelkalk/Keuper facies shift in the Central European Basin: Implications from the type-section of the Erfurt Formation (Lower Keuper, Triassic) and basin-wide correlations. International Journal of Earth Sciences.

[CR26] Franz M, Kaiser SI, Fischer J, Heunisch C, Kustatscher E, Luppold FW, Berner U, Röhling H-G (2015). Eustatic and climatic control on the Upper Muschelkalk Sea (late Anisian/Ladinian) in the Central European Basin. Global and Planetary Change.

[CR27] Geyer OF, Gwinner MP (2011). Geologie von Baden-Württemberg.

[CR28] Goldhammer RK, Dunn PA, Hardie LA (1990). Depositional cycles, composite sea-level changes, cycle stacking patterns, and the hierarchy of stratigraphic forcing: Examples from Alpine Triassic platform carbonates. Geological Society of America Bulletin.

[CR30] Hagdorn H, Wisshak MW, Löffler S-B, Schulbert C, Freiwald A (2006). Upper Muschelkalk of Crailsheim (Baden-Württemberg, SW Germany). 5th International bioerosion workshop programme & abstracts.

[CR32] Immenhauser A (2009). Estimating palaeo-water depth from the physical rock record. Earth-Science Reviews.

[CR33] Koehrer BS, Heymann C, Prousa F, Aigner T (2010). Multiple-scale facies and reservoir quality variations within a dolomite body: Outcrop analog study from the Middle Triassic, SW German Basin. Marine and Petroleum Geology.

[CR34] Kostic B, Aigner T (2004). Sedimentary and poroperm anatomy of shoal-water carbonates (Muschelkalk, South-German Basin): An outcrop-analogue study of inter-well spacing scale. Facies.

[CR72] Kozur H (1974). Biostratigraphie der germanischen Mitteltrias. Freiburger Forschungshefte C.

[CR35] Loreau J-P, Purser BH, Purser BH (1973). Distribution and ultrastructure of holocene ooids in the Persian Gulf. The Persian Gulf. Holocene carbonate sedimentation and diagenesis in a shallow epicontinental sea.

[CR36] Madritsch H (2015). Geology of central Northern Switzerland: Overview and some key topics regarding Nagra’s seismic exploration of the region. Swiss Bulletin for Applied Geology.

[CR37] Marchant R, Ringgenberg Y, Stampfli G, Birkhäuser P, Roth P, Meier B (2005). Paleotectonic evolution of the Zürcher Weinland (northern Switzerland), based on 2D and 3D seismic data. Ecologae Geologicae Helvetiae.

[CR38] Matysik M (2016). Facies types and depositional environments of a morphologically diverse carbonate platform: A case study from the Muschelkalk (Middle Triassic) of Upper Silesia, southern Poland. Annales Societatis Geologorum Poloniae.

[CR39] Menning M, Gast R, Hagdorn H, Käding K, Simon T, Szurlies M, Nitsch E (2005). Zeitskala für Perm und Trias in der Stratigraphischen Tabelle von Deutschland 2002, zyklostratigraphische Kalibrierung der höheren Dyas und Germanischen Trias und das Alter der Stufen Roadium bis Rhaetium 2005. Newsletters on Stratigraphy.

[CR40] Merki PJ (1961). Der Obere Muschelkalk im östlichen Schweizer Jura. Eclogae Geologicae Helvetiae.

[CR41] Molina JM, Ruiz-Ortiz PA, Vera JA (1997). Calcareous tempestites in pelagic facies (Jurassic, Betic Cordilleras, Southern Spain). Sedimentary Geology.

[CR42] Nagra (2001). Sondierbohrung Benken - Untersuchungsbericht. Nagra Technischer Bericht NTB 00-01.

[CR43] Palermo D, Aigner T, Nardon S, Blendinger W (2010). Three-dimensional facies modelling of carbonate sand bodies: Outcrop analog study in an epicontinental basin (Triassic, southwest Germany). American Association of Petroleum Geologists Bulletin.

[CR71] Palermo D, Aigner T, Seyfang B, Nardon S (2012). Reservoir properties and petrophysical modelling of carbonate sand bodies: Outcrop analogue study in an epicontinental basin (Triassic, Germany). Geological Society, London, Special Publications.

[CR44] Parrish JT (1993). Climate of the supercontinent Pangea. The Journal of Geology.

[CR45] Paul W, Sauer KFJ, Schnetter M (1971). Die trias. Die Wutach.

[CR46] Pérez-López A, Pérez-Valera F (2012). Tempestite facies models for the epicontinental Triassic carbonates of the Betic Cordillera (southern Spain). Sedimentology.

[CR47] Pietsch JS, Wetzel A, Jordan P (2016). A new lithostratigraphic scheme for the Schinznach Formation (upper part of the Muschelkalk Group of northern Switzerland). Swiss Journal of Geosciences.

[CR48] Pomar L (2001). Types of carbonate platforms: A genetic approach. Basin Research.

[CR49] Pomar L, Hallock P (2008). Carbonate factories: A conundrum in sedimentary geology. Earth Science Reviews.

[CR50] Pomar L, Ward WC (1999). Reservoir-scale heterogeneity in depositional packages and diagenetic patterns on a reef-rimmed platform, Upper Miocene, Mallorca, Spain. AAPG Bulletin.

[CR51] Pöppelreiter MC, Simone A, Hoetz G (2003). Reservoir characteristics of intracontinental carbonate ramp deposits: Upper Muschelkalk, Middle Triassic, NE Netherlands. Netherlands Journal of Geosciences.

[CR52] Ramseyer K, Fischer J, Matter A, Eberhardt P, Geiss J (1989). A cathodoluminescence microscope for low intensity luminescence. Journal of Sedimentary Research.

[CR53] Read J (1985). Carbonate platform models. American Association of Petroleum Geologists Bulletin.

[CR54] Ruf M, Aigner T (2004). Facies and poroperm characteristics of a carbonate shoal (Muschelkalk, South German Basin): A reservoir analogue investigation. Journal of Petroleum Geology.

[CR55] Schauer M, Aigner T (1997). Cycle stacking pattern, diagenesis and reservoir geology of peritidal dolostones, Trigonodus-Dolomite, Upper Muschelkalk (Middle Triassic, SW-Germany). Facies.

[CR56] Schwarz, M. (1985). Räumlicher und zeitlicher Ablauf der Sedimentation im Oberen Hauptmuschelkalk (Trias) von Südwestdeutschland. In *Arbeiten aus dem Institut für Geologie und Paläontologie an der Universität Stuttgart N.F.* (Vol. 81, pp. 11–50).

[CR57] Shinn EA (1983). Tidal flat environment. American Association of Petroleum Geologists Memoir.

[CR58] Strasser A (1984). Black-pebble occurrence and genesis in Holocene carbonate sediments (Florida Keys, Bahamas, and Tunisia). Journal of Sedimentary Research.

[CR59] Strasser A (1986). Ooids in Purbeck limestones (lowermost Cretaceous) of the Swiss and French Jura. Sedimentology.

[CR60] Strasser A (2018). Chapter three: Cyclostratigraphy of shallow-marine carbonates—Limitations and opportunities. Stratigraphy & Timescales.

[CR61] Stratsky S, Morard A, Möri A (2016). Harmonising the lithostratigraphic nomenclature: Towards a uniform geological dataset of Switzerland. Swiss Journal of Geosciences.

[CR62] Szulc J (2000). Middle Triassic evolution of the northern Peri-Tethys as influenced by early opening of the Tethys. Annales Societatis Geologorum Poloniae.

[CR63] Thury, M., Gautschi, A., Mazurek, M., Müller, W.H., Naef, H., Pearson, F.J., et al. (1994). *Technical report 93*-*01. Geology and hydrogeology of the crystalline basement of Northern Switzerland*. Wettingen: Nagra.

[CR64] Tucker M, Marshall J (2004). Diagenesis and geochemistry of Upper Muschelkalk (Triassic) buildups and associated facies in Catalonia (NE Spain): A paper dedicated to Francesc Calvet. Geologica Acta.

[CR65] Tyson RV, Pearson TH (1991). Modern and ancient continental shelf anoxia: An overview. Geological Society Special Publication.

[CR66] Vail PR, Audemard F, Bowman SA, Eisner PN, Perez-Cruz C, Einsele G, Ricken W, Seilacher A (1991). The stratigraphic signatures of tectonics, eustasy and sedimentology: An overview. Cycles and events in stratigraphy.

[CR67] Warnecke M, Aigner T (2019). Asymmetry of an epicontinental basin: Facies, cycles, tectonics and hydrodynamics—The Triassic Upper Muschelkalk, South Germanic Basin. The Depositional Record.

[CR68] Warnecke M, Aigner T (2019). Influence of subtle paleo-tectonics on facies and reservoir distribution in epeiric carbonates: Integrating stratigraphic analysis and modelling (U. Muschelkalk, SW Germany). Sedimentary Geology.

[CR69] Warren JK (2006). Evaporites: Sediments, resources and hydrocarbons.

[CR70] Ziegler PA (1990). Geological Atlas of Western and Central Europe.

